# Mapping Motor Neuron Vulnerability in the Neuraxis of Male SOD1^G93A^ Mice Reveals Widespread Loss of Androgen Receptor Occurring Early in Spinal Motor Neurons

**DOI:** 10.3389/fendo.2022.808479

**Published:** 2022-02-22

**Authors:** Victoria M. McLeod, Mathew D. F. Chiam, Nirma D. Perera, Chew L. Lau, Wah Chin Boon, Bradley J. Turner

**Affiliations:** ^1^ Florey Institute of Neuroscience and Mental Health, University of Melbourne, Parkville, VIC, Australia; ^2^ Perron Institute for Neurological and Translational Science, Queen Elizabeth Medical Centre, Nedlands, WA, Australia

**Keywords:** androgen receptor, ALS, motor neurons, neurodegeneration, sexual dimorphism

## Abstract

Sex steroid hormones have been implicated as disease modifiers in the neurodegenerative disorder amyotrophic lateral sclerosis (ALS). Androgens, signalling *via* the androgen receptor (AR), predominate in males, and have widespread actions in the periphery and the central nervous system (CNS). AR translocates to the cell nucleus when activated upon binding androgens, whereby it regulates transcription of target genes *via* the classical genomic signalling pathway. We previously reported that AR protein is decreased in the lumbar spinal cord tissue of symptomatic male SOD1^G93A^ mice. Here, we further explored the changes in AR within motor neurons (MN) of the CNS, assessing their nuclear AR content and propensity to degenerate by endstage disease in male SOD1^G93A^ mice. We observed that almost all motor neuron populations had undergone significant loss in nuclear AR in SOD1^G93A^ mice. Interestingly, loss of nuclear AR was evident in lumbar spinal MNs as early as the pre-symptomatic age of 60 days. Several MN populations with high AR content were identified which did not degenerate in SOD1^G93A^ mice. These included the brainstem ambiguus and vagus nuclei, and the sexually dimorphic spinal MNs: cremaster, dorsolateral nucleus (DLN) and spinal nucleus of bulbocavernosus (SNB). In conclusion, we demonstrate that AR loss directly associates with MN vulnerability and disease progression in the SOD1^G93A^ mouse model of ALS.

## Introduction

Amyotrophic lateral sclerosis is a progressive and fatal neurodegenerative disorder characterised by the loss of motor neurons (MNs) (1). ALS is defined by the loss of both upper and lower motor neurons. MNs are found throughout the length of the neuraxis with axonal processes spanning the peripheral nervous system to distant effector muscles. Glutamatergic upper motor neurons (UMN), referred to as corticospinal motor neurons (CSMNs), have cell bodies located in layer V of motor cortex. In humans, these large projection neurons are known as Betz cells, forming monosynaptic connections directly on lower motor neurons (LMN) in the brainstem and spinal cord. In mice, cortical to brainstem/spinal connectivity is indirect *via* interneurons (2). Cholinergic LMNs have cell bodies located in discrete brainstem clusters, also known as cranial nerve (CN) nuclei, and throughout the spinal cord ventral horn, known as spinal MNs.

Populations of somatic motor neurons show resistance to degeneration in ALS patients and mouse models. Two well established examples of this include, brainstem MNs innervating the extraocular muscles, and Onuf’s nucleus, innervating the pelvic-perineal muscles controlling urinary and faecal continence and sexual function (3–6). MNs are also differentially susceptible to degeneration based on their target fibre type innervation. The alpha (α)-MNs forming fast-twitch, fast-fatigable motor units are more vulnerable, compared to slow-twitch, fatigue-resistant motor units (7). The gamma (γ)-MNs which innervate the intrafusal muscle fibres, are resistant in ALS (8). Understanding the differences in molecular signatures of these MNs and the mechanisms of vulnerability are of great therapeutic interest in ALS.

To date, there has been little exploration into how sex steroid hormones may influence MN vulnerability. It is known that clinical heterogeneity in ALS stems from the site of onset and subsequent patterns of spread (9, 10). Sex-based differences in site of onset are now well established in clinical ALS (11). Bulbar onset occurs in one third of classical ALS patients and is characterised by loss of brainstem LMNs supplying oropharyngeal muscles. It is associated with a faster disease progression compared to limb/spinal onset (12) and is more common in females (11). Conversely, limb onset, which accounts for approximately two thirds of classical ALS cases, is more common in males where the male:female ratio can approach 2:1 in sporadic ALS (12). Respiratory onset, resulting from loss of phrenic MNs within the cervical spinal cord, occurs in 3-5% of cases and carries a poor survival prognosis with male bias (9, 13). The ALS variant, flail arm syndrome, resulting from degeneration in cervical LMNs innervating the upper limbs, also has a high male:female ratio of 4:1 (13). Understanding a potential relationship between sex hormone action and MN vulnerability could be beneficial in understanding the mechanisms of sexual dimorphism inherent in ALS.

AR is a nuclear steroid hormone receptor, activated by androgen binding, to initiate the classical AR signalling pathway, resulting in transcriptional regulation of target genes. Upon binding testosterone or dihydrotestosterone (DHT), AR protein conformation is stabilised (14), and the complex dimerises (15) and translocates to the nucleus where it binds to androgen response elements on DNA of target genes. A hypothesis that loss or dysfunction in AR may play a role in ALS was first proposed in 1980 (16), however, findings supporting an association are not well established. An original exploration into AR distribution, assessing tritium-labelled DHT binding, reported high AR binding in the α-MNs of the lower thoracic and lumbar spinal cord, and in some CN brainstem MN populations, excluding those resistant in ALS (17). Later, Ogata et al. reported that AR immunoreactivity was evident in the resistant MNs of CNs III, IV and VI, as well as Onuf’s nucleus, challenging the theory that AR absence conferred resistance in ALS (18). The discovery that an expansion mutation in the AR gene was responsible for LMN degeneration in spinal bulbar muscular atrophy (SBMA) (19), highlighted AR abundance in MNs and their selective vulnerability to aggregating disease proteins. The primary mechanism in SBMA is induction of nuclear AR aggregation upon binding of androgens. However, evidence also suggests a loss in AR function may contribute to disease pathogenesis (20, 21). Although, this gain of function mutation shares similar pathogenic mechanisms with ALS, a potential overlap in AR dysfunction in MNs remains unclear.

Here, we provide a comprehensive analysis of AR expression in MN populations vulnerable in the endstage SOD1^G93A^ mouse. Importantly, we mapped AR expression throughout the motor neuraxis of male mice, observing a profound loss of AR immunoreactivity within MNs of SOD1^G93A^ mice, with spinal and bulbar populations highly expressing AR being preserved. Our assessment of MN populations in the SOD1^G93A^ mouse revealed that spinal MNs in the lumbar spinal cord showed greatest vulnerability, followed by cervical MNs. CSMNs and the majority of brainstem MN populations were spared, other than the trigeminal and facial nuclei which showed mild degeneration. Further analysis revealed that lumbar MNs in SOD1^G93A^ mice showed loss of nuclear AR, which was apparent at pre-symptomatic age, prior to any overt MN loss. To our knowledge, this is the first evidence directly associating AR loss with MN vulnerability and disease progression in the SOD1^G93A^ mouse model of ALS.

## Materials and Methods

### Animals and Tissue Collection

All animal experiments were conducted in accordance with the Australian National Health and Medical Research Council published Code of Practice and the ARRIVE Guidelines (22). Approval was granted by Florey Institute of Neuroscience and Mental Health Animal Ethics Committee (approval number 15-060-FINMH) to conduct this project. Transgenic SOD1^G93A^ mice (B6.Cg-Tg(SOD1*^G93A^)1Gur/J line, stock number 004435, Jackson Laboratory) were bred on a C57BL/6J background and group housed at the Florey Institute of Neuroscience and Mental Health Core Animal Services under standard 12 h light-dark conditions with access to standard rodent chow and water *ad libitum*. The clinical symptoms of this SOD1 transgenic mouse have been described (23). Briefly, mice develop clinical symptoms from P90 which are usually clearly evident by P120, and include impaired hindlimb splaying when suspended, hindlimb weakness with impaired gait and weight loss. For motor neuron counts and AR expression mapping, SOD1^G93A^ males were aged to clinical endstage. This was defined as advanced but incomplete paralysis of the hindlimbs or complete hemiparalysis in either hindlimb, and/or a decline in body condition resulting in a cumulative loss of 20% peak body weight. In the current study, the average age of endstage was 160 ± 12 days for SOD1^G93A^ males. Tissue from non-transgenic (wildtype) littermates was collected at P150. We selected this endstage time point to observe maximal cell degeneration in motor neuron populations outside of the lumbar ventral horn which is the most vulnerable population in the SOD1^G93A^ mouse. Additional cohorts of mice age P60 (presymptomatic) and P120 (symptomatic) were used for lumbar tissue collection. Mice were killed by administration of sodium pentobarbitone (100 mg/kg, i.p.) followed by cardiac perfusion with 0.1 M PBS then 4% paraformaldehyde. Dissected brains and spinal cords were post-fixed for 2-4h, rinsed and cryoprotected in a sucrose gradient of 10, 20 and 30% sucrose in 0.1 M PB over 5 days. Tissue was frozen in isopentane cooled over dry ice and stored at -80°C prior to analysis.

### Immunohistochemistry—DAB Staining for ChAT

Brains and spinal cords were embedded in Tissue-Tek O.C.T embedding media (Sakura Finetek, CA) and cryosectioned at 20 µm in a 1 in 10 series onto poly-L-lysine coated glass slides. Antigen retrieval was performed by baking slides for 2 h at 98 °C in 10 mM citric acid, pH 6.0. Sections were blocked for 15 minutes in 0.5% hydrogen peroxide in PBS and 1 h at room temperature in 10% normal donkey serum in 0.3% Triton X-100 containing PBS. Slides were incubated in goat anti-ChAT primary antibody (1:200, Millipore, Cat# AB144P, RRID : AB_2079751) in 6% donkey serum:0.3% Triton X-100 containing PBS, at 4 °C for 48 h. Donkey anti-Goat IgG HRP-conjugate secondary antibody (1:500, Thermo Fisher Scientific, Cat# A16005, RRID : AB_2534679) was added to slides and incubated for 2 h at room temperature. DAB colorimetric reaction was performed using SignalStain^®^ DAB Substrate Kit (CST, Cat# 8059) according to manufacturer’s instructions.

### Immunohistochemistry—Fluorescent Staining for AR

Brains and spinal cords were cryosectioned using a Leica CM1860 cryostat at 20 µm in a 1 in 10 series onto poly-L-lysine coated glass slides. A subset of forebrains were cut as 50 µm free-floating and stored at -20 °C in cryoprotectant. Antigen retrieval was performed as outlined above. Sections were blocked in 10% normal donkey serum 0.3% Triton-X containing PBS for 1 h at room temperature (1% Triton-X was used for free floating sections). Endogenous avidin-biotin blocking was performed according to the manufacturer’s protocol (Endogenous Avidin/Biotin Blocking Kit, Abcam, Cat# ab64212). Slides were incubated in the following primary antibodies prepared in SignalStain^®^ Antibody Diluent (CST, Cat# 8112) for 48 h at 4°C: rabbit anti-AR (1:200, Abcam, cat# ab133273, RRID : AB_11156085), rat anti-Ctip2 (1:500, Abcam Cat# ab18465, RRID : AB_2064130), chicken anti-MAP2 (1:400, Abcam Cat# ab5392, RRID : AB_2138153), goat anti-ChAT (1:200), mouse anti-NeuN (1:1000, Millipore Cat# MAB377, RRID : AB_2298772) and mouse anti-SMI-32 (1:1000, Biolegend, cat# 801701, RRID : AB_ 2564642). A biotin amplification step was performed to increase AR detection where slides were incubated for 2 h at room temperature in donkey biotinylated anti-rabbit antibody (1:200, Jackson ImmunoResearch Cat# 711-065-152, RRID : AB_2340593). Slides were incubated in the following secondary antibodies for 2 h at room temperature: streptavidin Alexa Fluor^®^-488 (1:200, Jackson ImmunoResearch Cat# 016-540-084, RRID : AB_2337249), anti-chicken F(ab’)_2_ fragment Cy™3 (1:200, Jackson ImmunoResearch, Cat# 703-166-155, RRID : AB_2340364), anti-rat Alexa Fluor^®^-647 (1:200, Jackson ImmunoResearch, Cat# 712-605-153, RRID : AB_2340694), anti-goat DyLight^®^-550 (1:200, Thermo Fisher Scientific Cat# SA5-10087, RRID : AB_2556667) and anti-mouse F(ab’)_2_ fragment Alexa Fluor^®^-647 (1:200, Jackson ImmunoResearch Labs, Cat# 715-606-151, RRID : AB_2340866). Hoechst 33342 was used as a nuclear stain (ThermoFisher, Australia).

### Image Acquisition and Analysis

Chromogenic-stained sections were imaged on a Leica DM LB2 microscope (Leica Microsystems, Germany). For brainstem cranial nerve counts, nuclei regions were captured using 20x objective tiled scan, and where possible 3 sections were counted and averaged to give single hemisphere counts for each mouse. Counts for CNs X and XII represent both hemispheres due to their medial location and the merging of hemispheres. A count from n=6 mice was averaged for each genotype. For spinal cord cervical (C1-8) and lumbar (L1-5) regions, ChAT-stained motor neurons were counted in both hemispheres of the ventral horn under 20x objective, and averaged across 28-33 sections to give a count per mouse. A ventral horn count from n=5 mice was averaged for each genotype. In cranial nerves V and VII and spinal cord sections the smaller γ-MNs, were distinguished from larger α-MNs predominantly by their size, their more rounded cell morphology and more intense ChAT-staining. A retrospective comparison of MN sizes was performed and fit to Gaussian distribution. The optimal threshold to distinguish α- and γ-MNs based on size, as determined by the intersection of respective Gaussian distributions, was as follows: 300µm^2^ and 234 µm^2^ for WT and SOD1^G93A^ spinal MNs, respectively; 222µm^2^ and 241 µm^2^ for WT and SOD1^G93A^ CN V MNs, respectively; and 200µm^2^ and 212 µm^2^ for WT and SOD1^G93A^ CN VII MNs, respectively. To obtain phrenic MN counts, sections containing the phrenic motor column were identified by anatomical location, appearing as a MN cluster within an inter-medio-lateral position of the ventral horn of C3-5. All identifiable MNs sections were counted to provide an average count across 6 sections. The spinal nucleus of the bulbocavernosus (SNB), ventral medial nucleus (VMN), dorsolateral nuclus (DLN) and retrodorsolateral nucleus (RDLN) were located anatomically within L6 region of the lumbar spinal cord. All sections were counted and sum reported per mouse, before being averaged across n=5 mice per genotype. All counting was performed with investigator blinded to genotype.

For endstage mapping studies, fluorescent images were acquired on a Zeiss Axio 780 confocal microscope (Carl Zeiss AG, Germany). The location of the M1 motor cortex was guided by several anatomical landmarks including the corpus callosum, alignment of the lateral ventricles and anterior commissure and appearance of the third ventricle. For quantification, 1 µm Z-stack images of the M1 cortex from 20 µm slide-mounted sections were acquired using 20x objective, at Bregma 1.10, 0.86 and 0.62. The area containing M1 layer V was traced using Ctip2 density as an indicator of cortical layers, and CSMN were identified as large, pyramidal shaped neurons, staining positive for MAP2 and nuclear Ctip2. Representative regions of interest (ROI) for counting layer V CSMNs and cortical layer II/III neurons are presented in **Supplementary Material** (**Figures S1–S3**). CSMNs were counted manually by two independent counters blinded to genotype and reported as number per unit of area. Counts were then averaged across n=5 mice per genotype. For AR quantification in layer V, 50 µm free floating sections were used, and for cranial nerve nuclei, 20 µm slide-mounted sections were used. Exposure parameters were optimised for each ROI in brain, brainstem and spinal cord to the brightest AR expressing MNs within an image stack (typically this was in WT mice as these had much higher signal). Image acquisition settings and ROI were kept consistent for data collection across all mice and genotypes. Z-stack images were acquired at 1 µm intervals and from an average of n=3 mice and manually counted on Zen 2.5 lite software programme (Carl Zeiss). For quantitative analysis of AR nuclear staining intensity relative to cytoplasm, this was conducted in P60 and P120 lumbar tissue. Single Z-plane images of α-MNs in L2-5 spinal cord were acquired on a Leica SP8 confocal microscope under 40x objective with experimenter blinded to genotype. Mean grey values were determined for the nuclear area and cytoplasmic area of individual motor neurons using Leica Application Suite X (RRID : SCR_013673) and an average of ~35 neurons were analysed per mouse lumbar.

### Data and Statistical Analyses

CSMNs, ChAT positive and AR positive CN MNs, and spinal MNs were analysed by unpaired Student’s t-test comparing WT and SOD1^G93A^ mice. For M1 layer AR^+^ nuclei; α and γ MN subpopulation counts; P60 and P120 comparisons of AR staining intensity and AR positive nuclei, data were analysed by two-way ANOVA. Sidak’s multiple comparison test was performed to compare WT and SOD1^G93A^ counterparts, where F-value indicated a significant genotype effect (P<0.05). All analyses were performed using GraphPad Prism 8.0 software (San Diego, CA, USA) and data is presented as mean ± SEM.

## Results

### SOD1^G93A^ Mice Showed Regional Loss of AR Expression Within the Motor Cortex With No Loss in Layer V Projection Neuron Number at endstage

**Figure 1:**
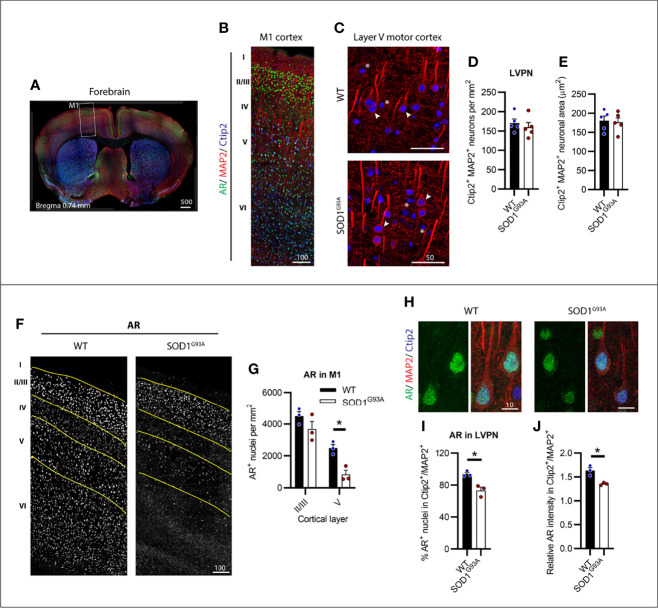
Layer V projection neurons (LVPN) expressed high levels of AR and were preserved in endstage SOD1^G93A^ male mice. **(A)** Identification of primary motor cortex (M1) in mouse forebrain coronal sections. **(B)** Layers of the M1 cortex identified by Ctip2 staining and MAP2 projection neurons with AR nuclei observed throughout layers II-VI. **(C)** LVPN, including corticospinal motor neurons (CSMN), were identified as larger MAP2 positive cell bodies and apical dendrites with Ctip2 positive nuclei (indicated by arrows), smaller round Ctip2 nuclei (indicated by asterisk) were not counted as CSMN. **(D)** Quantification of Ctip2^+^/MAP2^+^ positive LVPN in WT and SOD1^G93A^ mice per unit area of layer V and **(E)** somal area. Mean ± SEM, n = 5 mice. **(F)** Representative images of AR^+^ staining by motor cortex layer with **(G)** quantification of positive nuclei per unit area within layers II/III and V. *P < 0.05 significantly different to WT male by two-way ANOVA with Sidak’s multiple comparisons test comparing genotype. **(H)** Representative images of AR^+^ nuclei identified in LVPN with **(I)** quantification of AR^+^ population and **(J)** the mean intensity of AR nuclear signal relative to mean intensity of Ctip2^+/^MAP2^+^ neuron. *P < 0.05 significantly different to WT male by unpaired t-test. Mean ± SEM, n = 3 mice. Scale bars = µm units.

We identified CSMNs within M1 cortex of male mice using Ctip2 and MAP2 markers (**Figures 1A, B**). Ctip2 is a nuclear marker most prominent in layer V pyramidal neurons (LVPNs), including CSMNs in the motor cortex (24). MAP2 was used to determine the overall neuronal architecture to select for CSMNs based on their large cell body and apical dendrite. We analysed several regions of motor cortex, tracing layer V to determine the CSMN density within this select region (**Figure S1**). SOD1^G93A^ mice showed no detectable loss in large Ctip2^+^/MAP2^+^ LVPNs, presumed to represent the CSMN population (**Figures 1C, D**). Likewise, no evidence of somal atrophy (**Figures 1C, E**) or dendrite retraction (**Figure S2**), indicative of cellular degeneration, was observed in SOD1^G93A^ mice at disease endstage. Within the motor cortex of P150 WT mice, AR appeared in all cortical layers (**Figures 1B, F**). Using Ctip2 nuclear marker alongside AR we were able to delineate layers II/III and V of the cortex (**Figure S3**) which were observed to show the highest intensity AR nuclei. AR nuclei appeared markedly reduced in all layers, except II/III, in the SOD1^G93A^ mouse motor cortex (**Figure 1F** and **Figure S3**). This was confirmed by AR^+^ nuclear density analysis within layer II/II and V (**Figure 1G**) revealing a significant effect of both genotype (F_1,8 =_ 14.11; P=0.0056) and cortical layer (F_1,8 =_ 54.36; P<0.0005) on detectable AR. AR nuclei were most abundant in WT layer II/III neurons which was maintained in SOD1^G93A^ mice (P=0.2218). In contrast, a 67% reduction in AR detectable within layer V neurons occurred in SOD1^G93A^ mice compared to WT (**Figure 1G**). AR was highly expressed in the nuclei of almost all LVPNs of WT mice (**Figure 1H** **;** **Table 1**), with SOD1^G93A^ males displaying a reduction in LVPNs with detectable nuclear AR staining (94% vs. 73% for WT and SOD1^G93A^ mice, respectively; **Figure 1I**). This was paralleled by a similar reduction in the nuclear immunoreactivity of AR in SOD1^G93A^ LVPNs (**Figure 1J**). In summary, CSMNs in male mice expressed high levels of AR and were resistant to degeneration in SOD1^G93A^ mice. These neurons displayed a moderate loss in AR by endstage disease.

**Table 1:** Characterisation of AR staining intensity in the motor neuron subtypes and clusters within the central nervous system of male wild-type mice.

Motor neuron population	Location	MN Classification	% AR positive (Mean ± SD)	Staining intensity	% change from WT
LVPN/CSMN	M1 cortex	Upper	94 ± 3	++/+++	ns
III (Oculomotor nucleus)	Brainstem	Lower—Somatic	49 ± 9	+	ns
IV (Trochlear nucleus)	Brainstem	Lower—Somatic	48 ± 15	+	ns
VI (Abducens nucleus)	Brainstem	Lower—Somatic	16 ± 13	-/+	ns
V (Trigeminal nucleus)	Brainstem	Lower—Branchial	68 ± 5	++	41 *
VII (Facial nucleus)	Brainstem	Lower—Branchial	69 ± 15	++	22 *
X (Dorsal nucleus of vagus nerve)	Brainstem	Lower—Visceral	86 ± 3	+++	ns
XII (Hypoglossal nucleus)	Brainstem	Lower—Somatic	74 ± 9	+/++	ns
Amb (Nucleus ambiguus)	Brainstem	Lower—Branchial/Visceral	91 ± 5	+++	ns
Cervical ventral horn	C1-8 spinal cord	Lower—Somatic	69 ± 5	++	54 *
Phrenic nuclei	C3-4 spinal cord	Lower—Somatic	79 ± 15	++	53 *
Lumbar ventral horn	L1-5 spinal cord	Lower—Somatic	81 ± 3	++	67 *
RDLN	L6 spinal cord	Lower—Somatic	75 ± 4	++	41 *
DLN	L6 spinal cord	Lower—Somatic	97 ± 6	+++	ns
VMN	L6 spinal cord	Lower—Somatic	100	++	60 *
SNB	L6 spinal cord	Lower—Somatic	100	+++	ns

LVPN, layer V pyramidal neuron; CSMN, corticospinal motor neuron; RDLN, retrodorsolateral nucleus; DLN, dorsolateral nucleus; VMN, ventral medial nucleus; SNB, spinal nucleus of the bulbocavernosus.

*P < 0.05 indicated a significant decrease compared to WT; ns, not significantly different by unpaired t-test.

### Somatic Motor Neurons of the Brainstem Cranial Nerves III (Oculomotor), IV (Trochlear) and VI (Abducens) Displayed Low AR Expression Without Degeneration in SOD1^G93A^ Mice

**Figure 2:**
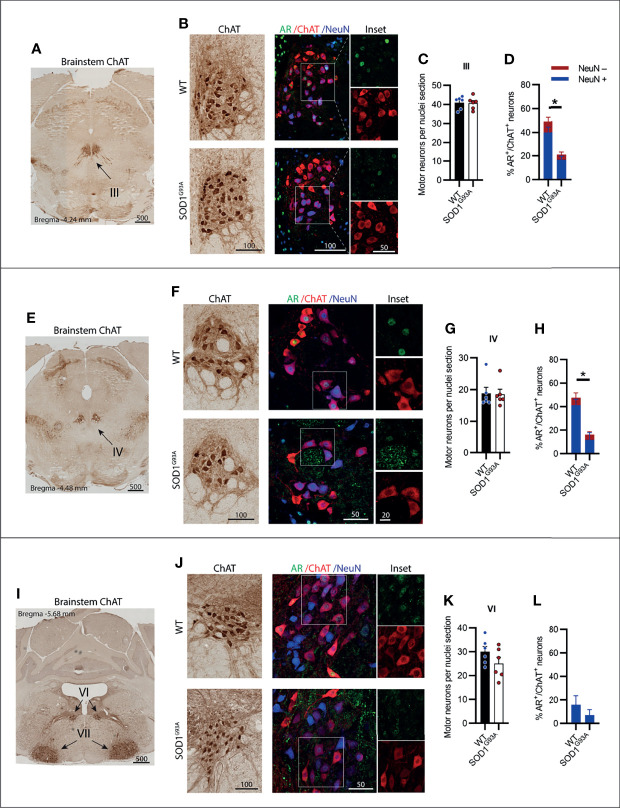
Brainstem cranial nerve III (Oculomotor), IV (Trochlear) and VI (Abducens) motor neurons innervating the extraocular eye muscles were not lost in SOD1^G93A^ mice and displayed low level AR expression. **(A)** ChAT staining to identify cranial nerve III nuclei located medially within the midbrain. **(B)** Chromogenic ChAT labelling of nerve III motor neurons in WT and SOD1^G93A^ alongside immunofluorescent labelling of AR nuclei and NeuN within this ChAT positive population. **(C)** Quantification of chromogenic ChAT positive neurons within cranial nerve III sections of WT and SOD1^G93A^ mice and **(D)** quantification of AR positive staining with NeuN positive and negative proportions. **(E)** ChAT staining to identify cranial nerve IV nuclei located medially within the midbrain. **(F)** Chromogenic ChAT labelling of nerve IV motor neurons in WT and SOD1^G93A^ alongside immunofluorescent labelling of AR nuclei and NeuN within this population. **(G)** Quantification of chromogenic ChAT positive neurons within cranial nerve IV sections of WT and SOD1^G93A^ mice and **(H)** quantification of AR positive staining with NeuN positive and negative proportions in nerve IV motor neurons. **(I)** ChAT staining to identify cranial nerve VI nuclei located medially within the midbrain. **(J)** Chromogenic ChAT labelling of nerve VI in WT and SOD1^G93A^ alongside immunofluorescent labelling of AR nuclei and NeuN within this population. **(K)** Quantification of chromogenic ChAT positive neurons within cranial nerve IV sections of WT and SOD1^G93A^ mice and **(L)** quantification of AR positive staining with NeuN positive and negative proportions. Mean ± SEM, n = 6 mice for ChAT cell counts, n = 3 for AR quantification. *P < 0.05 significantly different to WT male by unpaired t-test. Scale bars = µm units.

MNs innervating the extraocular eye muscles are known to be preserved in SOD1^G93A^ mice (3). The oculomotor nerve or cranial nerve III (CN III) is the most rostrally located of the MN nuclei in the brainstem, located within the midbrain in the position of the superior colliculus (**Figure 2A**). These MNs innervate most of the muscles controlling eye and eyelid movement. No change in ChAT^+^ MN numbers within CN III nuclei were observed in SOD1^G93A^ mice (**Figures 2B, C**). A significant reduction in AR^+^ nuclear expression was evident in MNs from SOD1^G93A^ mice (**Figures 2B, D**) with AR detectable in 21 ± 4% of MNs, compared to 49 ± 5% in WT mice (**Table 1**). To explore AR expression among MN subpopulations, we used NeuN staining to identify α-MNs, distinguishable from γ-MN which have low abundance or non-detectable NeuN. The loss in AR detected in SOD1^G93A^ mice occurred similarly across both MN populations within CN III (**Figure 2D**). The α-MNs predominate in CN III nuclei (73% of ChAT^+^ MNs) and AR was slightly more abundant in this subpopulation with 55% staining positive, compared to 31% of the total γ-MN in WT mice. The trochlear nerve or cranial nerve IV (CN IV) is located caudolaterally to the CN III nuclei with the two cranial nuclei often grouped together (**Figure 2E**). The CN IV innervates a single extraocular muscle of the eye controlling internal rotation. Likewise, no loss in CN IV MNs was observed in SOD1^G93A^ mice (**Figures 2F, G**), although a significant reduction is nuclear AR was evident, with only 16 ± 4% of MNs having detectable AR, compared to 48 ± 7% of MNs in WT mice (**Figures 2F, H**; **Table 1**). The representation of AR staining by MN subpopulations in CN IV was similar to that of CN III MNs in both genotypes. Finally, the abducens nerve or cranial nerve VI (CN VI) is the third CN to innervate the extraocular eye muscles, controlling lateral eye movement, with MNs located medially within the brainstem between the pons and medulla junction (**Figure 2I**). Again, no loss in CN VI MNs occurred in SOD1^G93A^ mice (**Figures 2J, K**) with very low detection of nuclear AR in both genotypes (**Figure 2L** and **Table 1**). Taken together, the cluster of CNs innervating the eye muscles show weak AR expression detectable in less than 50% of MNs. No degeneration was evident in these MNs, despite SOD1^G93A^ mice having reduced AR compared to WT animals.

### Branchial Motor Neurons of the Brainstem Cranial Nerves V (trigeminal) and VII (Facial) Displayed Moderate AR Expression Levels and Were Lost in Endstage SOD1^G93A^ Mice

**Figure 3:**
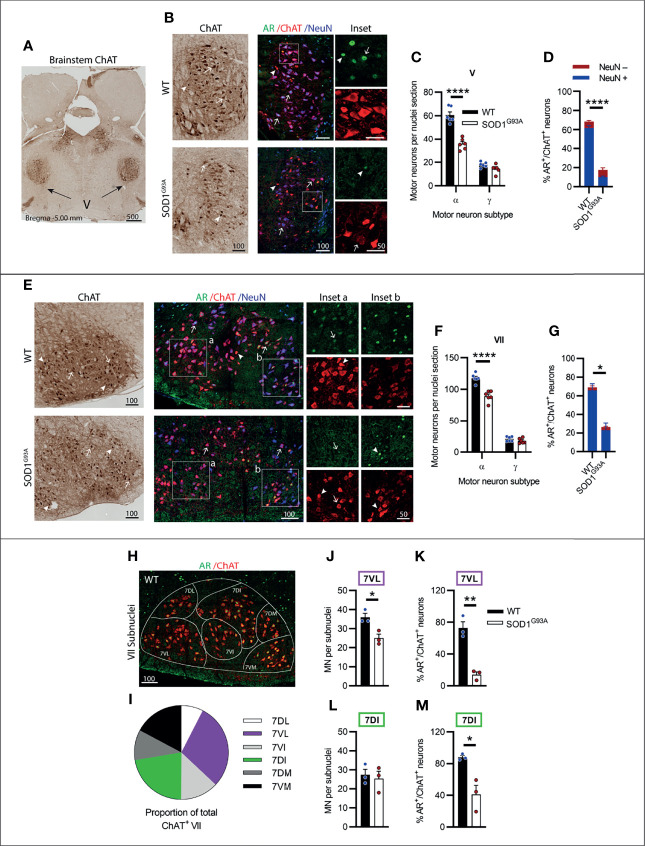
Brainstem cranial nerve V (Trigeminal) and VII (Facial) nuclei expressing moderate level of AR showed cell loss in endstage SOD1^G93A^ mice. **(A)** ChAT staining to identify cranial nerve V nuclei located laterally within the hindbrain. **(B)** Chromogenic ChAT labelling of nerve V in WT and SOD1^G93A^ alongside immunofluorescent labelling of AR nuclei within this ChAT positive population. **(C)** Quantification of chromogenic ChAT positive neurons within cranial nerve V sections of WT and SOD1^G93A^ mice based on size and morphology to distinguish alpha and gamma subpopulations alongside **(D)** quantification of AR positive staining with NeuN positive and negative proportions. Representative alpha and gamma motor neurons identified by arrows and arrow heads, respectively. **(E)** Chromogenic ChAT labelling of nerve VII in WT and SOD1^G93A^ alongside immunofluorescent labelling of AR nuclei and NeuN within this ChAT positive population. AR staining shows greater intensity in the medial subnucleus (inset b) compared to laterally located subnuclei (inset a). **(F)** Quantification of chromogenic ChAT positive neurons within cranial nerve VII sections of WT and SOD1^G93A^ mice alongside **(G)** quantification of AR positive staining with NeuN positive and negative proportions. **(H)** Division of CN VII motor neurons into subnuclei: dorsolateral (DL), ventrolateral (VL), ventral intermediate (VI), dorsal intermediate (DI), dorsomedial (DM) and ventromedial (VM) and **(I)** proportion of MNs making up each subnucleus. **(J)** Quantification of ChAT MNs in CN VII subnuclei 7VL in WT vs SOD1^G93A^ mice alongside **(K)** quantification of AR positive nuclei staining. **(L)** Quantification of ChAT MNs in CN VII subnuclei 7DI in WT vs SOD1^G93A^ mice alongside **(M)** quantification of AR positive nuclei staining. ****P < 0.0001 significantly different to WT male by two-way ANOVA with Sidak’s multiple comparisons test comparing genotype for ChAT quantification; mean ± SEM, n = 6. *P < 0.05, **P < 0.01, ****P < 0.0001 significantly different to WT male by unpaired t-test for AR quantification and subnuclei quantification, mean ± SEM, n=3. Scale bars = µm units.

The trigeminal nerve or cranial nerve V (CN V) is the first of the larger multi-branched CNs with MN nuclei located in the brainstem pons, laterally to CN VI (**Figure 3A**). CN V innervates eight muscles involved in chewing, biting and swallowing functions (25). In ChAT-stained sections, γ-MNs were delineated from α-MNs primarily based on their smaller size (**Table S1, Figure S4** in **Supplementary Material**), often exhibiting a rounded morphology and higher ChAT signal intensity. Genotype (F_1,20 =_ 56.81, P<0.0001) and MN subtype (F_1,20 =_ 287, P<0.0001) had a significant effect on ChAT^+^ neuron numbers in CN V. SOD1^G93A^ mice lost ~41% of CN V α-MNs (**Figures 3B, C**), while γ-MNs (making up ~23 of CN V MNs in WT mice) were preserved (**Figure 3C**). In fluorescent stained tissue, the γ-MNs were also identified by a lack of NeuN (26); based on this delineation 82% of ChAT MNs in WT mice were positive for NeuN and 18% were negative for NeuN. In the SOD1^G93A^ mouse CN V, a reduction in nuclear AR levels was evident, with only 18 ± 2% of MNs having AR^+^ nuclear staining, compared to 68 ± 3% of MNs in WT mice (**Figure 3D**). This loss was preferentially in NeuN^+^ α-MNs, with only 16% of the remaining population staining positive for AR compared to 75% of α-MNs in WT mice. In SOD1^G93A^ mice, 20% of NeuN^-^ γ-MNs were AR^+^, which is a slight reduction compared to WT γ-MNs where 37% were AR^+^.

The facial nerve or cranial nerve VII (CN VII) is the largest CN with motor neuron nuclei located in the ventral aspect of the pons (**Figure 2I**) innervating the muscles of the face controlling facial expression (27). Genotype (F_1,20 =_ 39.3, P<0.0001) and MN subtype (F_1,20 =_ 11.38, P<0.0001) had a significant effect on ChAT^+^ neuron numbers in CN VII. In SOD1^G93A^ mice, ~22% of CN VII α-MNs were lost by endstage disease with no loss in γ-MN (which represented 15% of total CV VII MNs in WT mice; **Figures 3E, F** and **Table S1**). In fluorescent stained tissue, there was regional variation in ChAT and NeuN staining intensity across MN populations (**Figure 3E**) and NeuN was also present in the nuclei of most small MNs. In the CNs, γ-MNs can appear similar in size to smaller α-MN (28), making subtype delineation more difficult. In WT mice, NeuN^+^ staining was detected in 93% of ChAT MNs with variable cytoplasmic and/or nuclear intensity. Therefore, we quantified AR staining in the total MN population. Again, a significant reduction in AR levels was evident in SOD1^G93A^ mouse CN VII, with only 26 ± 8% of MNs observed to have AR^+^ nuclear staining, compared to 69 ± 9% of ChAT^+^ neurons in WT mice (**Figures 3E, G**; **Table 1**).

Within the CN VII, clusters of lower expressing AR^+^ MNs were evident in the ventral-lateral zone (**Figure 3E**, inset a), while subnuclei in the dorsal-medial zones exhibited some more intensely stained AR^+^ ChAT^+^ neurons (**Figure 3E**, inset b). To explore further the regional specific variation in MN vulnerability and AR expression, the CN VII was divided into 6 subnuclei, which are named relative to their anatomical location within CN VII nucleus (**Figure 3H**), also reflecting topographical innervation of the facial muscles (29). We selected two CN VII subnuclei for further investigation; the lateral subnucleus (7VL) innervating the nasolabialis muscles, and the dorsal intermediate (7DI) subnucleus innervating the facial eye area (**Figures 3H, I**). In SOD1^G93A^ mice, there was a 30% loss in MN within the 7VL subnucleus (**Figure 3J**), whereas MNs in the 7DI subnucleus were preserved (**Figure 3L**). A reduction in AR^+^ MNs was evident in both subnuclei of SOD1^G93A^ mice, although this was more pronounced in the 7VL region, which was reduced from 72% to 14% AR^+^ MNs in WT vs. SOD1^G93A^, respectively (**Figure 3K**), compared to 88% vs. 41% in the 7DI region (**Figure 3M**). In summary, the CNs V and VII were vulnerable to MN degeneration in SOD1^G93A^ mice and showed a prominent loss in AR expression.

### Visceral Motor Neurons of the Brainstem Vagus Nerve Complex, X (Vagus) and Amb (Nucleus ambiguus), Expressed High AR Levels and Were Preserved in SOD1^G93A^ Mice

**Figure 4:**
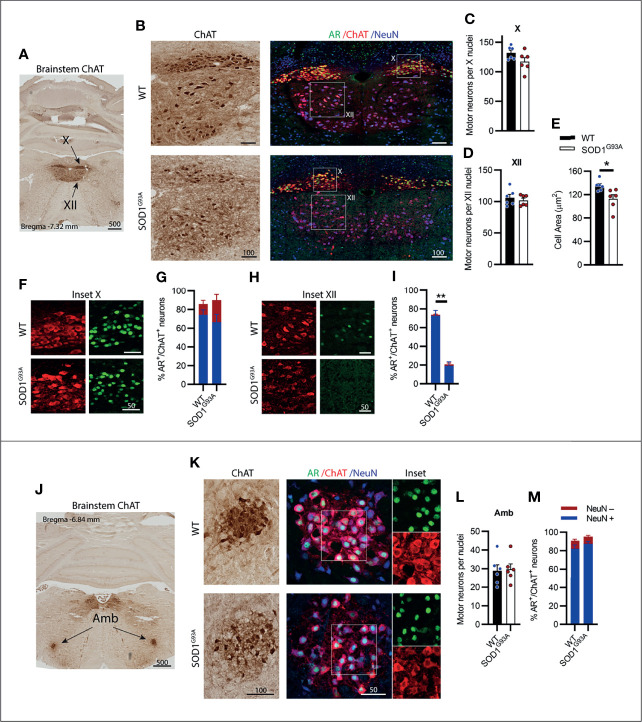
Brainstem cranial nerve X (Vagus) and ambiguus (Amb) motor neurons expressing high level of AR; and cranial nerve XII (Hypoglossal) motor neurons expressing moderate level of AR did not show cell loss in endstage SOD1^G93A^ mice. **(A)** ChAT staining to identify cranial nerve X and XII nuclei located medially within the hindbrain. **(B)** Chromogenic ChAT labelling of nerve X and XII in WT and SOD1^G93A^ alongside immunofluorescent labelling of AR nuclei and NeuN within these ChAT positive populations. **(C)** Quantification of chromogenic ChAT positive neurons in WT and SOD1^G93A^ CN X and **(D)** CN XII, alongside **(E)** cell body area in XII MNs. **(F)** AR staining shows greater intensity in the CN X nuclei (inset X) with **(G)** quantification of AR^+^ nuclei staining in NeuN^+/-^ ChAT MNs. **(H)** AR staining in XII nuclei (inset XII) with **(I)** quantification of AR^+^ nuclei staining in NeuN^+/-^ ChAT MNs. **(J)** ChAT staining to identify Amb nuclei located laterally within the hindbrain. **(K)** Chromogenic ChAT labelling of Amb nucleus in WT and SOD1^G93A^ alongside immunofluorescent labelling of AR nuclei and NeuN within this ChAT positive population. **(L)** Quantification of chromogenic ChAT positive neurons within Amb sections of WT and SOD1^G93A^ mice alongside **(M)** quantification of AR^+^ nuclei staining in NeuN^+/-^ ChAT MNs. Mean ± SEM, n=6 mice for ChAT cell counts, n=3 for AR quantification. *P < 0.05, **P < 0.01 significantly different to WT male by unpaired t-test. Scale bars = µm units.

A CN nucleus rich in AR expression is the dorsal nucleus of the vagus nerve, also known as cranial nerve X (CN X), with MNs located in the medulla oblongata of the brainstem below the fourth ventricle (**Figure 4A**). These MNs differ from the other CNs in that their projections form visceral efferents innervating the parasympathetic autonomic ganglia of the thorax and abdomen. The CN X MNs showed a tightly packed and rounded cell morphology with consistent low level NeuN expression (**Figure 4B**). No decrease in ChAT^+^ cell count was observed in SOD1^G93A^ mice (**Figure 4C**). AR was highly expressed in the nuclei of CN X MNs irrespective of genotype, detectable in 86 ± 2% of MNs in WT and 90 ± 10% of MNs SOD1^G93A^ (**Figures 4F, G**). The nucleus ambiguus (Amb) is a group of motor neurons situated in the medullary reticular formation of the brainstem (**Figure 4J**) and has a branchial MN component innervating the muscles of the palate, pharynx and larynx, as well as a visceral MN component innervating the heart (30). Amb MNs were preserved in SOD1^G93A^ mice at endstage disease (**Figures 4K, L**). SOD1^G93A^ mice showed high nuclear AR expression, detectable in 95 ± 2% of ChAT^+^ neurons within the Amb nucleus, similar to WT mice exhibiting detectable nuclear AR in 91 ± 3% (**Figures 4K, M** and **Table 1**) of MNs. Together, the CN X and Amb MNs represented a unique class of MNs having some visceral innervation. They also displayed the highest AR expression among the CN MN populations and showed no evidence of AR loss or degeneration in SOD1^G93A^ mice.

### Somatic Motor Neurons of the Brainstem Cranial Nerve XII (hypoglossal) Expressed Low to Moderate AR levels and Showed Early Signs of Degeneration in SOD1^G93A^ Mice

Situated immediately ventral to the CN X is the hypoglossal nerve or cranial nerve XII (CN XII), which innervates the muscles of the tongue. No loss in ChAT^+^ CN XII MNs was observed in SOD1^G93A^ mice (**Figures 4B, D**). Further analysis performed on the morphology of the CN XII MNs revealed a 17% loss in soma volume in SOD1^G93A^ mice (**Figure 4E**). A clear delineation between α- and γ-MNs was not evident in the CN XII MNs and NeuN^+^ was detectable in 97% of ChAT^+^ MNs. CN XII MNs expressed a low to moderate level of AR^+^ which was decreased in SOD1^G93A^ mice, where 21 ± 5% of MNs had AR^+^ nuclear staining (**Figure 4I**), compared to 74 ± 5% of MNs in WT mice (**Figures 4H, I**; **Table 1**). In conclusion, the CN XII MNs did not show any cell loss in SOD1^G93A^ mice, similar to earlier described somatic CN MNs innervating the eye muscles. These CN XII MNs displayed a low AR level and showed early morphological evidence of degeneration.

### Motor Neurons in the Cervical Spinal Cord Showed Consistent AR Expression Levels and Increased Susceptibility to Degeneration

**Figure 5:**
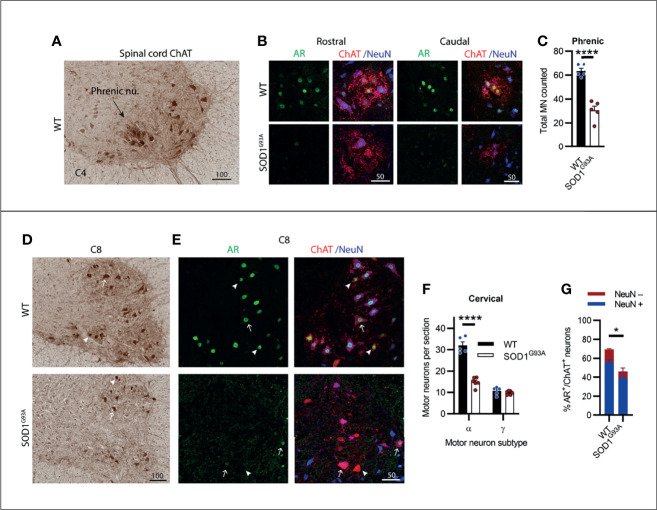
Cervical spinal cord motor neurons expressed consistent moderate levels of AR and showed extensive neurodegeneration in endstage SOD1^G93A^ mice. **(A)** ChAT staining in C4 spinal cord of WT mice to identify the phrenic nucleus located within the ventral horn of WT mice. **(B)** Representative immunofluorescent labelling of AR and NeuN within this ChAT positive MN population from a rostrally and caudally located section containing the phrenic motor column. **(C)** Quantification of chromogenic ChAT positive neurons in the phrenic nuclei of C3-5 of WT and SOD1^G93A^. Mean ± SEM, n=5 mice. ****P < 0.0001 significantly different to WT male by unpaired t-test. **(D)** ChAT staining in level C8 spinal cord alongside **(E)** immunofluorescent labelling of AR and NeuN. Alpha and gamma motor neurons identified by arrows and arrow heads, respectively. **(F)** Quantification of chromogenic ChAT positive neurons in the ventral horns of cervical spinal cord regions C1-8 of WT and SOD1^G93A^ using size and morphology to distinguish alpha and gamma subpopulations. Mean ± SEM, n = 5 mice. ****P < 0.0001 significantly different to WT male by two-way ANOVA with Sidak’s multiple comparisons test comparing genotype. **(G)** Quantification of AR positive staining with NeuN positive and negative proportions. *P < 0.05, significantly different to WT male by unpaired t-test for AR quantification, mean ± SEM, n = 3. Scale bars = µm units.

The cervical spinal cord contains several motor columns innervating the neck and mastoid musculature (C1-5) and the upper limbs (C5-8). It also contains the phrenic motor column, extending from C3-C5, innervating the diaphragm which we have located and shown in **Figure 5A**. In SOD1^G93A^ mice, 53% of phrenic MNs were lost by endstage disease (**Figures 5B, C**) and AR staining was notably reduced in the remaining MN pool. The ChAT^+^ cervical spinal MNs showed a clear delineation between α- and γ-MN populations, where γ-MNs had small, rounded morphology (**Figure 5D**; **Figure S4; Table S1**) and an absence in cytoplasmic and nuclear NeuN (**Figure 5E**). Genotype (F_1,18 =_ 82.8, P<0.0001) and MN subtype (F_1,18 =_ 177, P<0.0001) had a significant effect on ChAT^+^ neuron numbers in the cervical ventral horn. In SOD1^G93A^ mice, 54% of α-MNs were lost through C1-C8, while there was no change in γ-MNs comparable to WT mice (**Figure 5F**). A significant reduction in AR levels was observed in SOD1^G93A^ cervical MNs, with 46 ± 6% of remaining MNs having AR^+^ nuclear staining (compared to 69 ± 3% in WT mice), although with markedly reduced intensity (**Figures 5E, G**). In remaining NeuN^+^ α-MNs, 55% had low level AR nuclear staining (compared to 69% of α-MNs in WT mice) and AR was mostly absent in the NeuN^-^ γ-MN population with only 6% having detectable AR signal (compared to 58% of NeuN^-^ γ-MNs in WT mice). In summary, spinal MNs in the cervical region showed pronounced cell loss in SOD1^G93A^ mice, much greater than the neurodegeneration described in brainstem MNs.

### Motor Neurons in the Lumbar Spinal Cord Were Most Vulnerable to Degeneration in SOD1^G93A^ Mice With Clusters of Highly AR Expressing Neurons Being Preserved

**Figure 6:**
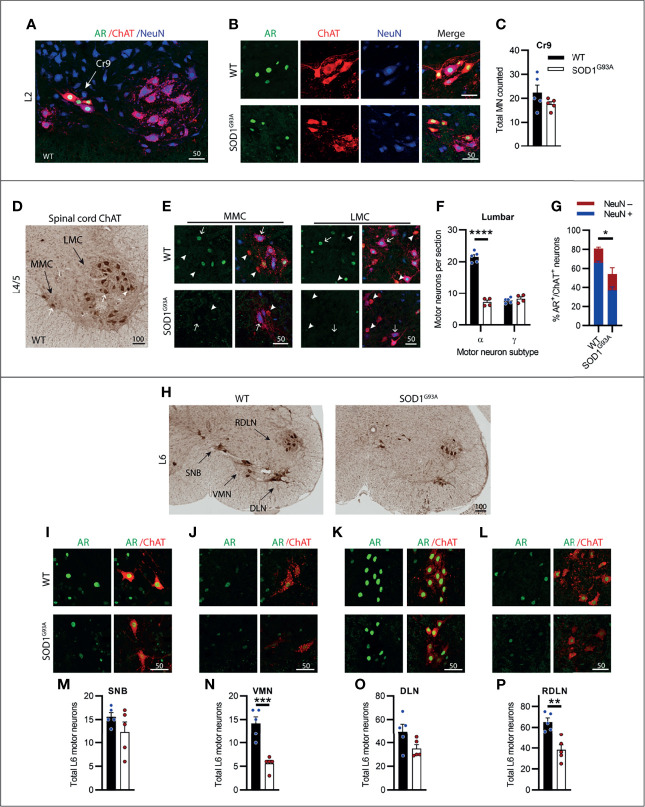
Lumbar spinal cord motor neurons expressed heterogenous levels of AR and showed the greatest loss in endstage SOD1^G93A^ mice with evidence of high AR expressing resistant populations. **(A)** AR, ChAT and NeuN staining of from L2 spinal cord ventral horn of WT mice to identify a subpopulation of high AR expressing cremaster (Cr9) motor neurons. **(B)** Representative images of Cr9 motor neurons present in both WT and SOD1^G93A^ within rostral lumbar regions which show mixed NeuN expression. **(C)** Quantification of Cr9 MN populations in fluorescent-stained images of L1-2 of WT and SOD1^G93A^ male mice. Mean ± SEM, n = 5 mice. **(D)** ChAT staining in level L4/5 of spinal cord of WT mice with motor neurons clustered into a median motor column (MMC) and lateral motor column (LMC). **(E)** Immunofluorescent labelling of AR and NeuN in ChAT-stained motor neurons from the MMC (left panel) and LMC (right panel) regions of WT and SOD1^G93A^ spinal cord. Alpha and gamma motor neurons identified by arrows and arrow heads, respectively. **(F)** Quantification of chromogenic ChAT positive neurons in the ventral horns of lumbar spinal cord regions L1-5 of WT and SOD1^G93A^ using size and morphology to distinguish alpha and gamma subpopulations. Mean ± SEM, n = 5 mice. ****P < 0.0001 significantly different to WT male by two-way ANOVA with Sidak’s multiple comparisons test comparing genotype. **(G)** Quantification of AR positive staining with NeuN positive and negative proportions. Mean ± SEM, n = 3; *P < 0.05, significantly different to WT male by unpaired t-test. **(H)** Chromogenic ChAT staining in L6 spinal cord of WT and SOD1^G93A^ mice showing sexually dimorphic motor neuron populations including spinal nucleus of bulbocavernosus (SNB), ventromedial nucleus (VMN), dorsolateral nucleus (DLN) and retrodorsolateral nucleus (RDLN). Immunofluorescent labelling of AR in ChAT^+^ motor neuron nuclei within: **(I)** SNB, **(J)** VMN, **(K)** DLN and **(L)** RDLN of L6 male spinal cords of WT with SOD1^G93A^ with quantification of chromogenic ChAT counts provided below for **(M)** SNB, **(N)** VMN, **(O)** DLN and **(P)** RDLN. Mean ± SEM, n = 5 mice. **P < 0.01, ***P < 0.001 significantly different to WT male by unpaired t-test. Scale bars = µm units.

The lumbar spinal cord predominantly contains MNs innervating the lower limb and pelvis musculature. Most of the ChAT^+^ MNs are found in two motor columns within the ventral horn, the medial motor column (MMC) innervating axial muscles of posture and the larger lateral motor column (LMC) innervating the limbs. Genotype (F_1,18 =_ 130.6, P<0.0001) and MN subtype (F_1,18 =_ 112, P<0.0001) had a significant effect on ChAT^+^ neuron numbers in the lumbar ventral horn. We observed a high AR-containing cluster of MNs present in the early sections of the lumbar spinal cord located intermediate to the MMC and lateral to LMC motor nuclei (**Figure 6A**). These likely represented the cremaster (Cr9) MNs which innervate the cremaster reflex (31), a superficial reflex which serves a protective physiological function within of the testes of males. The Cr9 MNs did not undergo degeneration in SOD1^G93A^ mice (**Figure 6C**) and AR immunoreactivity was highly conserved in these neurons (**Figures 6B, C**). Conversely, MNs present in the MMC and LMC throughout L1-5 were the most vulnerable MN population in SOD1^G93A^ mice (**Figures 6D–F**) with 67% of the α-MNs lost through L1-L5 (**Figure 6F**) and no loss in γ-MNs (which made up 26% of the total ChAT^+^ ventral horn MN population in WT mice, **Table S1**). A significant reduction in AR levels was evident in SOD1^G93A^ mice, with 55 ± 9% of remaining MNs retaining AR nuclear staining, albeit at markedly reduced intensity, compared to 81 ± 2% of MNs in WT mice (**Figures 6E, G**). Consistent with that observed in cervical MNs, in SOD1^G93A^ mice the remaining NeuN^+^ lumbar α-MNs, showed very low level AR nuclear expression, detected in 55% of α-MNs (compared to 85% of α-MNs in WT mice; **Figure 6G**). In the NeuN^-^ γ-MN population from SOD1^G93A^ mice, 53% maintained detectable, but weaker nuclear AR (compared to 75% of γ-MN in WT mice).

The caudal section of the lumbar spinal cord running into the early sacral regions contains several sexually dimorphic MN populations which innervate the pelvic/perineal musculature in male mice. This includes the highly AR expressing spinal nucleus of the bulbocavernosus (SNB), located in the central area of L5-S1, and the dorsolateral nucleus (DLN) located, as the name suggests, in the dorsal lateral aspect of the ventral horn of L6-S1 (**Figure 6H**). In SOD1^G93A^ mice, neither of these nuclei exhibited significant MN loss, compared to WT mice (**Figures 6M, O**) and maintained high AR expression (**Figures 6I, K**) at endstage disease. The sparse neurons contained in the ventromedial nucleus (VMN; **Figures 6J, N**) and the monomorphic retrodorsolateral nucleus (RDLN; **Figures 6L, P**) were typical of the general lumbar MN population and showed significant reductions in ChAT^+^ counts in SOD1^G93A^ mice, with 60% loss in the VMN (**Figure 6N**) and 42% loss in the RDLN (**Figure 6P**). In SOD1^G93A^ mice, the intensity of AR staining in MNs was observed to be decreased in both VMN and RDLN, although we did not quantify expression levels. The results from the lumbar spinal cord when taken together, showed that the lumbar MNs were the most vulnerable population in SOD1^G93A^ mice and displayed the greatest cell loss in the neuraxis. AR staining intensity was also profoundly decreased in the surviving MNs, although a low level was still detectable in half of these MNs. The lumbar spinal cord also contained several MN populations with high AR expression which did not undergo degeneration in SOD1^G93A^ mice.

### AR Expression Was Decreased in Lumbar Motor Neurons of SOD1^G93A^ Male Mice From Pre-Symptomatic Age

**Figure 7:**
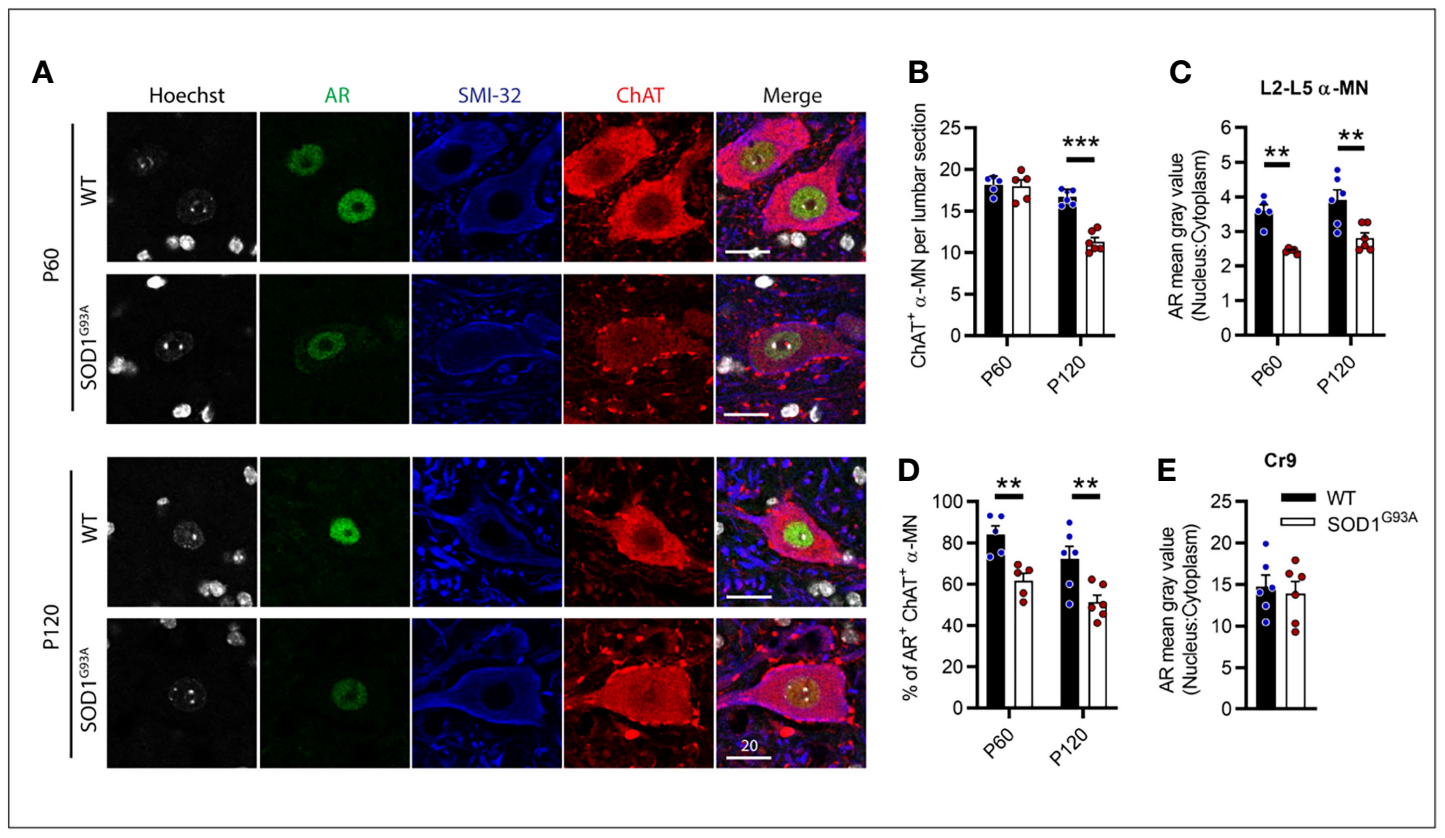
Androgen receptor nuclear staining was decreased in SOD1^G93A^ male mice lumbar motor neurons from pre-symptomatic (P60) age compared to wildtype mice. **(A)** AR nuclear staining identified in ChAT and SMI-32 positive alpha MNs at P60 and P120 ages, scale bar = 20 µm. **(B)** Quantification of ChAT positive alpha MNs per section in the lumbar spinal cord of P60 and P120, WT and SOD1^G93A^ mice alongside, **(C)** measured by mean grey value in L2-5 α MNs. **(D)** Percentage of ChAT positive α MNs with detectable nuclear AR staining. **(E)** Quantification of nuclear to cytoplasmic AR staining intensity the highly AR expressing cremaster (Cr9) population in L2 from P120 mice. Data represent mean ± SEM, n = 5-6 mice per group. **P < 0.01, ***P < 0.001, significantly different to WT male by two-way ANOVA with Sidak’s multiple comparisons test comparing genotype.

With AR expression noticeably diminished throughout MNs of the CNS, we further explored this loss within the vulnerable MNs of the lumbar spinal cord over the disease course in male SOD1^G93A^ mice. P60 was chosen as an early pre-symptomatic time prior to MN loss and clinical onset. This is also a typical time for treatment initiation in SOD1^G93A^ mouse studies. P120 was also chosen to explore AR as clinical symptoms are established at this time but prior to the rapid deterioration toward endstage paralysis. For these studies, α-MNs were identified by ChAT and SMI-32 positive cytoplasmic staining and were selected from ventral horn regions through L2-L5 (**Figure 7A**). L6 was excluded as this region contains high AR-expressing sexually dimorphic MN populations (32). Genotype (F_1,18 =_ 27.11, P<0.0001) and age (F_1,18 =_ 57.04, P<0.0001) had significant effects on α-MN numbers in the lumbar ventral horn (**Figure 7B**). At presymptomatic age (P60), SOD1^G93A^ mice did not show any MN loss (**Figure 7B**). Genotype had significant effects on nuclear AR intensity (F_1,18 =_ 32.8, P<0.0001; **Figure 7C**) and AR^+^ nuclear scoring in α-MNs (F_1,18 =_ 22.89, P<0.0001; **Figure 7D**). At P60, AR nuclear staining intensity was significantly reduced by approximately 30% in SOD1^G93A^ MNs compared to WT MNs (**Figures 7A, C**) with AR being detected in 60% of α-MNs (compared to 84% of α-MNs in WT mice; **Figure 7D**). At P120, WT mice MN count was comparable to P60 WT (P=0.1495), while SOD1^G93A^ mice showed a 32% loss in ChAT^+^ MNs compared to WT (**Figure 7B**). Age was not found to influence nuclear AR intensity, however, it did significantly contribute to AR^+^ nuclear scoring (F_1,18 =_ 6.016, P=0.0246; **Figure 7D**). Further analysis did not find a difference between P60 and P120 WT mice nuclear AR (P=0.1594). In the remaining α-MNs of P120 SOD1^G93A^ mice, AR nuclear staining intensity was reduced by 28% the level in WT mice (**Figures 7A, C**) with AR being detected in 50% of α-MNs (**Figure 7D**). By contrast, the highly expressing AR MN population, Cr9, showed ~3.8-fold higher AR staining intensity compared to other α-MNs, and was comparable between SOD1^G93A^ and WT at P120 (**Figure 7E**).

## Discussion

Determining what influences MN vulnerability in ALS is critical for developing therapeutic interventions. MNs in the motor cortex, brainstem and spinal cord diverge across multiple physiological and biochemical properties. These include synaptic connectivity, composition of effector targets, gene expression profiles and protein homeostasis (33–35). AR is known to be differentially expressed throughout the neuraxis and the role of androgen signalling within various MN populations remains poorly understood. We previously reported that AR protein expression and androgen metabolising enzyme, 5α-reductase type 2, were reduced in the lumbar spinal cords of symptomatic SOD1^G93A^ male mice (36). Building on this preliminary evidence, we hypothesised that AR may influence MN vulnerability in this ALS model. In the present study, we comprehensively mapped AR expression in the MNs of the male mouse motor system, and in parallel assessed MN loss in the SOD1^G93A^ model of ALS. All ALS vulnerable MN populations consistently showed moderate AR nuclear expression in WT counterparts, which was robustly downregulated in endstage SOD1^G93A^ male mice. In the lumbar spinal cord, this downregulation in nuclear AR appeared to be present from presymptomatic age, prior to MN loss.

### CSMNs in SOD1^G93A^ Mice and Cortical AR Expression

In sporadic and familial ALS patients, the large pyramidal Betz cells in layer V motor cortex show extensive vacuolisation of apical dendrites (37). Betz cells are synonymous with CSMNs in the mouse M1 cortex, however in mice, CSMNs are much smaller and difficult to identify (38). The clinical endstage in SOD1^G93A^ mice was determined by complete or hemiparalysis in the hindlimbs. At this stage, we did not identify a loss or change in the CSMNs. This contrasted with two studies in SOD1^G93A^ mice which reported significant losses in CSMNs. Ozdinler and colleagues reported up to 67% reduction in CSMNs in P120 SOD1^G93A^, compared to WT, with CSMNs identified by retrograde fluorogold tracer (39). CSMNs were reportedly decreased by 58% in P120 SOD1^G93A^ mice, when identified using GFP-expression driven by the UCHL1 promotor (40). Interestingly, in both cases there were parallel losses in subcerebral projection neurons in other cortical areas including the somatosensory cortex. While using axonally transported, retrograde labelling with either virus or tracer remains the gold standard for identifying CSMN populations, these may only capture a specific vulnerable pool of the MN population. Factors such as 1) the integrity of the corticospinal tract, spinal grey matter or muscle at injection location; 2) accuracy and timing of administration; 3) confinement of retrograde label to the target area, may impact the effectiveness of these labelling techniques. Additionally, the UCHL1 gene is known to be downregulated in sporadic ALS motor cortex (41), hence, it may be lost in a disease-progressive manner, as is the case in several other neurodegenerative disorders (42, 43). It is also possible that other cortical projection neurons were included in our count. In the mouse M1, corticospinal, corticothalamic, corticostriatal and corticocortical projections all emerge from layer V, with corticothalamic projection neurons showing similar size and locality to the corticospinal neurons (44). Taken together, accurate detection and quantification of CSMN loss in SOD1^G93A^ mice remains challenging, and no change was found in LVPNs projection neurons resembling CSMN in ~P160 endstage SOD1^G93A^ male mice in the current study.

AR signalling has been shown to regulate spine density in hippocampal pyramidal neurons (45) and attenuates dendritic atrophy in spinal MNs (46), in addition, providing neuroprotection to a range of neurons (47). Therefore, AR signalling may also play a role in supporting the health of CSMNs. In the present study we observed decreased AR staining in layer V of the motor cortex of SOD1^G93A^ mice, with moderately decreased nuclear AR in the larger LVPNs, representing CSMNs. With no evidence of CSMN degeneration in SOD1^G93A^ mice in the present study, this mouse model may not best reflect clinical ALS in which UMNs are also involved. It is possible that AR does not influence CSMNs, at least to the same extent as LMNs. In support of this, there is no sex bias in ALS presenting with predominantly UMN involvement (48), nor are UMNs involved in SBMA, a LMN disorder caused by an expansion mutation in AR (49).

We observed that AR staining was most abundant in the layer II/III neurons, with nuclear expression maintained in these neurons of SOD1^G93A^ mice. In rat cortex, AR was found to be present predominantly in the pyramidal neurons of layers II/III and V/VI in sensory and motor cortices (50), consistent with our findings in the mouse motor cortex. Retrograde tracer studies revealed that a large proportion of the layer V AR positive nuclei are associated with corticocortical projection neurons (50). Layer II/III neurons provide the major excitatory input to CSMN dendrites, including the thalamocortical inputs relaying cognitive and sensory information from other cortical areas (51). Cognitive impairment occurs in ~50% of ALS patients with 15-20% classified as having frontotemporal dementia (FTD) (52). ALS-FTD occurs more frequently in women with increasing age (48) and cognitive impairment with executive dysfunction is also more frequent in women (53). Androgens regulate executive function (54) and are protective against cognitive decline, with multiple reports of androgen-deprivation therapy being associated with increased dementia in prostate cancer patients (55). Taken together, this evidence supports androgens and AR influencing cognitive impairments in ALS more strongly than providing neurotrophic support to UMNs.

### Vulnerability of Brainstem Motor Neurons

We showed that CSMNs in SOD1^G93A^ do not degenerate by endstage disease. In the brainstem, we observed MN loss in several CN populations. MN loss was clearly evident in both trigeminal (CN V) and facial (CN VII) nuclei, in line with other SOD1 transgenic mouse models (3, 56). We observed no loss in hypoglossal CN XII MNs, however, these MN displayed soma volume loss, an early indicator of degeneration. This data is in line with ALS patients, where the CNs V, VII and XII are all affected in early stages of disease (34). We showed that the high AR expressing MNs, innervating viscera, exhibited no degeneration and maintained their nuclear AR in endstage SOD1^G93A^ mice. Conversely, the MNs of the oculomotor (CN III), trochlear (CN IV) and abducens (CN VI) all expressed AR at lower levels which was detectable in less than half of the MN population. Our data provides insight into the conflicting theories on the role of AR in MNs which may have previously over generalised a relationship between AR level and MN vulnerability across LMN populations. Early hypotheses suggested that AR presence coincided with MN vulnerability and resistant CN MNs showed AR absence or low levels (16, 17). Our findings are in support of this, whereby the LMNs which contained moderate AR levels were more likely to degenerate in SOD1^G93A^ mice, possibly being more susceptible to the loss in AR occurring throughout MNs of the neuraxis in disease. We observed this throughout the brainstem and spinal cord MNs, with low level AR populations in CNs III, IV, VI and XII showing no cell loss by endstage disease in SOD1^G93A^ mice. An alternative hypothesis proposed evidence that AR expression in MNs conferred resistance in ALS and this included CNs III, IV, VI and Onuf’s nucleus (18). We dispute high AR expression in the oculomotor nuclei, although our findings support a theory that subpopulations of MNs expressing a high level of nuclear AR do show resistance to degeneration, as well as retaining their AR levels in endstage disease.

Androgens are protective to injured brainstem MNs. Facial nerve MNs show evidence of AR-mediated neuroprotection in axotomy models; testosterone permanently rescued ~20% of MN in the postnatal hamster facial nerve axotomy model (57) and DHT transiently enhanced facial MN survival in the adult mouse facial nerve crush model (58). Testosterone improved regeneration of hypoglossal MNs following nerve crush injury in rats (59) with the tongue muscle being the primary target for testosterone-mediated neuroprotection following axotomy of the hypoglossal nerve (60). While these studies infer that androgens have neuroprotective actions against direct axonal damage in the CN nuclei, it is unknown if androgens confer the same protection against neurodegenerative MN death.

### Vulnerability of Spinal Motor Neurons

Similar to cranial nerve injury models, androgens promote spinal MN recovery following sciatic nerve crush injury in rats (61), with evidence supporting androgen-mediated neurite growth and axonal recovery well documented (62). The SOD1^G93A^ mouse model best replicates spinal MN pathology and the dying back phenomenon, which has been likened to an axonopathy, whereby loss in synaptic connectivity at the NMJ may be the initiating site of the disease (63). AR is enriched in cells at the NMJ (64) and presents as a potential target site for androgen neuroprotection. In endstage SOD1^G93A^ rodents, there is an amplification in activity of remaining phrenic MNs to maintain stimulation to the diaphragm (65). Testosterone improves diaphragm neurotransmission, reducing fatigue during repetitive firing (66). The loss of AR within MNs may further impair their ability to mount effective compensatory mechanisms to maintain NMJ integrity in ALS.

Alongside protecting the NMJ and axonal health, the dendritic arbor of MNs is critical in regulating synaptic input, excitation and supporting MN health (37, 67). The fast-fatigable motor units are most vulnerable in ALS, compared to slow and fatigue-resistant motor units and most cranial MNs, such as oculomotor. These vulnerable MNs tend to show a greater soma volume and increased dendritic complexity, compared to the resistant subtypes (33, 68, 69). Androgens provide neuroprotection against secondary dendritic atrophy induced by injury to surrounding somatic MNs (46, 70). In the SOD1^G93A^ mouse lumbar MNs, maladaptive dendrite morphology occurs as early as postnatally, with degeneration in dendrites evident by pre-symptomatic P60 age (68), a time we observed a reduced nuclear AR. While most MNs exhibit a decrease in AR in endstage SOD1^G93A^, larger spinal MNs with more extensive dendritic arborisations may be more susceptible to the impact of local androgen changes and AR downregulation.

Androgens such as testosterone and DHT bind to AR, prompting translocation of the complex into the nucleus, which also stabilises AR protein from degradation (14). In lumbar α-MNs, we report here a reduction in nuclear AR from as early as P60 prior to any cell death, and remaining diminished over disease course. The enzyme that primarily converts testosterone to the more potent DHT within androgen-responsive tissues, 5α-reductase type 2, is expressed in large pyramidal neurons in the brain and ventral horn MNs in male spinal cord (71, 72). We previously reported that transcript levels of this enzyme were selectively reduced in the spinal cord of symptomatic SOD1^G93A^ male mice, while being conserved in prostate tissue (36), and may be responsible for localising AR loss to MNs of the ventral horn and LVPNs in the brain. Further evidence is provided from ALS patients who have markedly reduced DHT concentrations in CSF, while free testosterone levels remain unchanged (73). DHT administration to male SOD1^G93A^ mice improved motor function and survival (74). The loss in nuclear AR evident early in disease course prior to any MN loss, may suggest antecedent mechanisms contributing to MN vulnerability, rather than a consequence of disease course.

In conclusion, a robust decrease in AR expression levels within MNs was evident throughout the CNS of male SOD1^G93A^ mice. In the lumbar α-MNs, a reduction in nuclear AR staining was evident prior to MN loss and may reflect earlier reports of dysregulated local androgen biosynthesis in the SOD1^G93A^ mouse. Unique subsets of MNs displaying high intensity AR did not appear to undergo degeneration in SOD1^G93A^ mice. We did not observe a universal correlation between AR expression and vulnerability in ALS through the CNS, likely reflecting the complexity and diversity across different MN populations and the mild influences of steroid hormone signalling on MN survival. Larger, more branched, spinal α-MN are potentially more sensitive to decreased AR signalling and further exploration of the functional impacts of altered AR in these MNs is warranted.

## Data Availability Statement

The raw data supporting the conclusions of this article will be made available by the authors, without undue reservation.

## Ethics Statement

The animal study was reviewed and approved by Florey Institute of Neuroscience and Mental Health Animal Ethics Committee.

## Author Contributions

Conceptualisation (VM), performed experiments (VM), analysed data (VM, MC), writing original draft (VM), review and editing (NP, CL, WCB, BT), provided supervision (CL, WCB, BT), funding acquisition (BT). All authors contributed to the article and approved the submitted version.

## Funding

Funding for this project was provided by the Australian NHMRC (Project Grants 1104295, 1104299), Stafford Fox Medical Research Foundation, MND Research Institute of Australia (Ted Dimmick Memorial MND Research Grant). VM was supported by a MND Research Institute of Australia PhD Scholarship Top-Up Grant. NP was supported by a MND Research Institute of Australia Postdoctoral Fellowship. BT was supported by a NHMRC-ARC Dementia Research Leadership Fellowship 1137024. The Florey Institute of Neuroscience and Mental Health acknowledge Victorian Government Operational Infrastructure Support.

## Conflict of Interest

The authors declare that the research was conducted in the absence of any commercial or financial relationships that could be construed as a potential conflict of interest.

## Publisher’s Note

All claims expressed in this article are solely those of the authors and do not necessarily represent those of their affiliated organizations, or those of the publisher, the editors and the reviewers. Any product that may be evaluated in this article, or claim that may be made by its manufacturer, is not guaranteed or endorsed by the publisher.

## Abbreviations

ALS, amyotrophic lateral sclerosis; AR, androgen receptor; CN, cranial nerve; CNS, central nervous system; CSF, cerebrospinal fluid; CSMN, corticospinal motor neuron; DHT, dihydrotestosterone; DLN, dorsolateral nucleus; ER, estrogen receptor; LMN, lower motor neurons; LVPN, layer V pyramidal neuron; MN, motor neuron; NMJ, neuromuscular junction; RDLN, retrodorsolateral nucleus; SBMA, spinal bulbar and muscular atrophy; SNB, spinal nucleus of the bulbocavernosus; SOD1, superoxide dismutase 1; UMN, upper motor neuron; VMN, ventral medial nucleus; WT, wildtype.
