# Supplementary material for: Mapping Motor Neuron Vulnerability in the Neuraxis of Male SOD1G93A Mice Reveals Widespread Loss of Androgen Receptor Occurring Early in Spinal Motor Neurons
Source: Front Endocrinol (Lausanne). 2022 Feb 22;13:808479. doi: 10.3389/fendo.2022.808479 (PMC8902593; doi:10.3389/fendo.2022.808479)
Supplement: Supplementary file 1 [file DataSheet_1.pdf]

# Supplementary Material

# Mapping motor neuron vulnerability in the neuraxis of male SOD1$^{G93A}$ mice reveals widespread loss of androgen receptor occurring early in spinal cord motor neurons

**Victoria M. McLeod[1], Mathew D.F. Chiam[1], Nirma D. Perera[1], Chew L. Lau[1], Wah C. Boon[1], Bradley J. Turner[1,2,*]**

[1]Florey Institute of Neuroscience and Mental Health, University of Melbourne, Parkville, VIC 3052, Australia

[2]Perron Institute for Neurological and Translational Science, Queen Elizabeth Medical Centre, Nedlands, WA 6150, Australia

*** Correspondence:**
Bradley Turner
bradley.turner@florey.edu.au

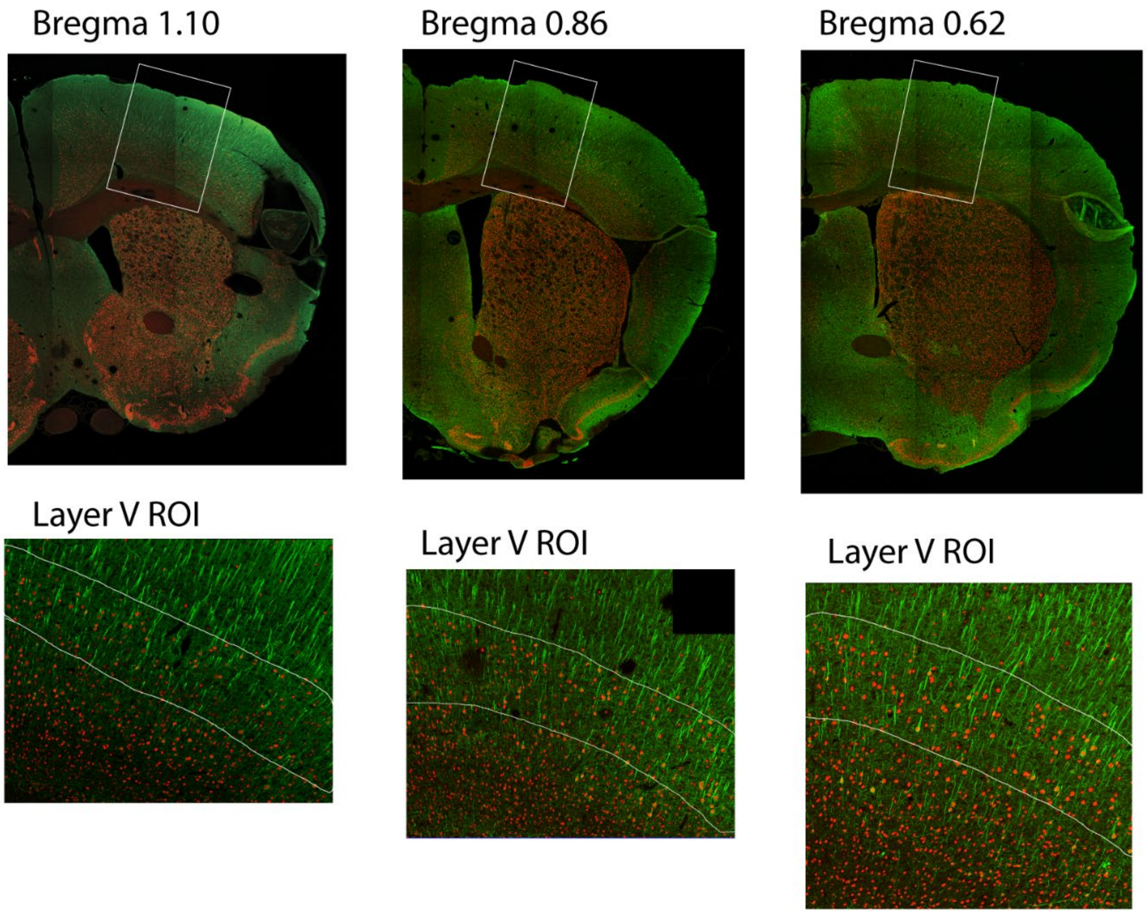


**Figure S1.** Identification of M1 cortex in different coronal sections of mouse forebrain with higher magnification image outlining the layer V region of interest (ROI) by white dotted line, delineated using Ctip2 (red) and MAP2 (green) immunohistochemistry.

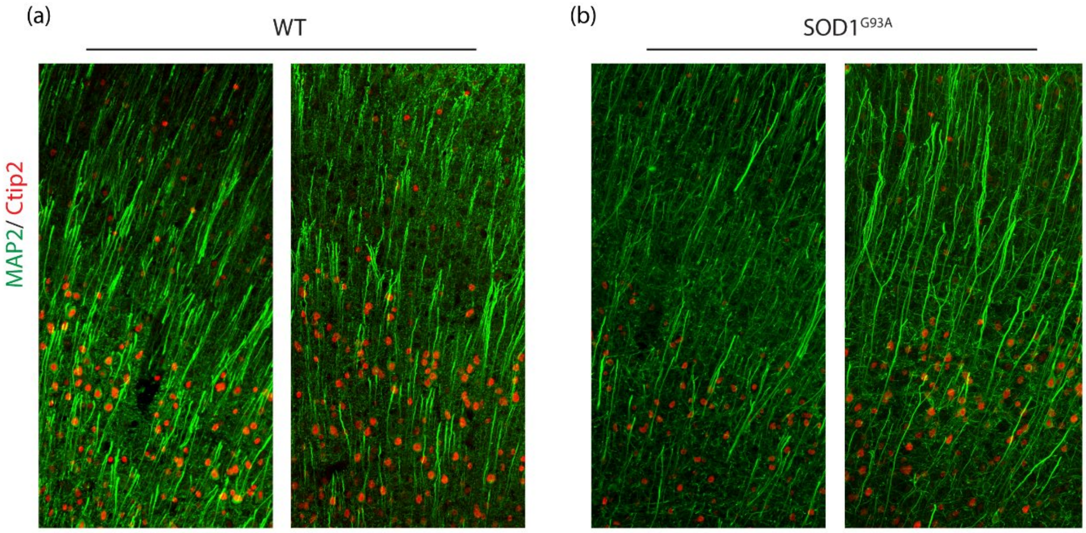


**Figure S2.** Immunohistochemical staining of MAP2 and Ctip2 in layers II-V of the M1 mouse cortex in (a) WT and (b) SOD1$^{G93A}$ mice. The density of apical dendrites (MAP2) projecting from the layer V projection neurons (Ctip2) do not appear to be diminished in the endstage SOD1$^{G93A}$ mice**.**

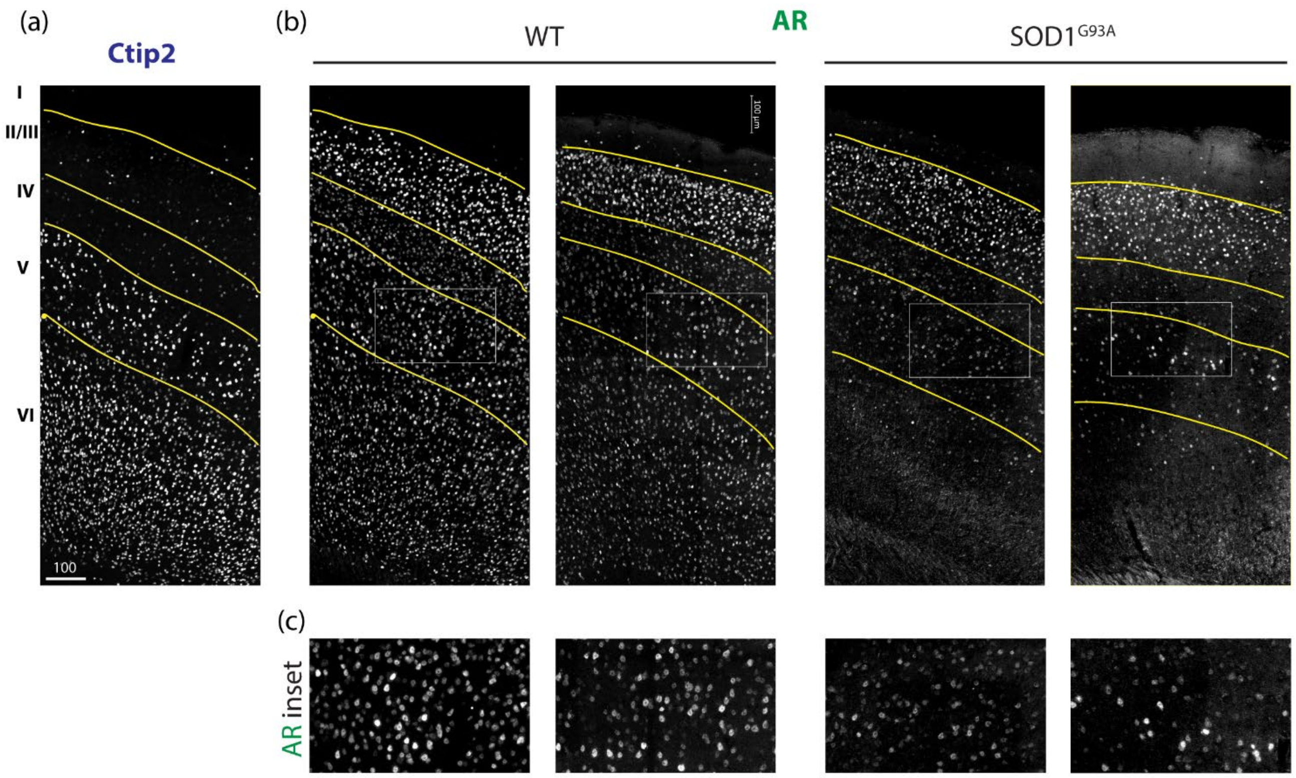


**Figure S3.** (a) Immunohistochemical staining of Ctip2 in the M1 cortex to delineate the lower cortical layers. (b) AR immunostaining overlayed with cortical layer borders (yellow lines) to indicate different regions of interest (ROI) for AR nuclear quantification. AR appears noticeably reduced in lower layers of the M1 including (c) layer V of the SOD1$^{G93A}$ mice compared to WT.

**Table S1. Assessment of motor neurons by size comparing two immunostaining methods**

| Population | Genotype | Detection Method | α-MN (μm$^2$) | γ-MN (μm$^2$) | % γ-MNs |
|---|---|---|---|---|---|
| Spinal | | | | | |
| Lumbar | WT | ChAT chromogenic | 622 ± 205 | 219 ± 71 | 26 ± 1.3 |
| | WT | ChAT/NeuN Fluoro | 763 ± 227 | 313 ± 68 | 23 ± 8 |
| | SOD1$^{G93A}$ | ChAT chromogenic | 363 ± 149 | 147 ± 27 | 53 ± 6.1 |
| | SOD1$^{G93A}$ | ChAT/NeuN Fluoro | 480 ± 141 | 277 ± 80 | 34 ± 8 * |
| Brainstem | | | | | |
| V | WT | ChAT chromogenic | 414 ± 138 | 136 ± 48 | 23 ± 3.8 |
| | WT | ChAT/NeuN Fluoro | 476 ± 148 | 199 ± 64 | 18 ± 1.2 |
| | SOD1$^{G93A}$ | ChAT chromogenic | 337 ± 117 | 142 ± 59 | 29 ± 5.4 |
| | SOD1$^{G93A}$ | ChAT/NeuN Fluoro | 337 ± 107 | 175 ± 64 | 36 ± 11 |
| VII | WT | ChAT chromogenic | 332 ± 97 | 105 ± 40 | 15 ± 2.5 |
| | WT | ChAT/NeuN Fluoro | 382 ± 113 | 178 ± 67 | 7.4 ± 3.1 * |
| | SOD1$^{G93A}$ | ChAT chromogenic | 269 ± 89 | 133 ± 58 | 17 ± 4.1 |
| | SOD1$^{G93A}$ | ChAT/NeuN Fluoro | 293 ± 91 | 139 ± 56 | 17 ± 0.9 |

* Significantly different to ChAT chromogenic detection method by unpaired t-test. Statistical analysis was only performed on % γ-MN in the MN pools counted as we consistently observed slightly lower area in the chromogenic sections compared to fluorescence across all MNs.

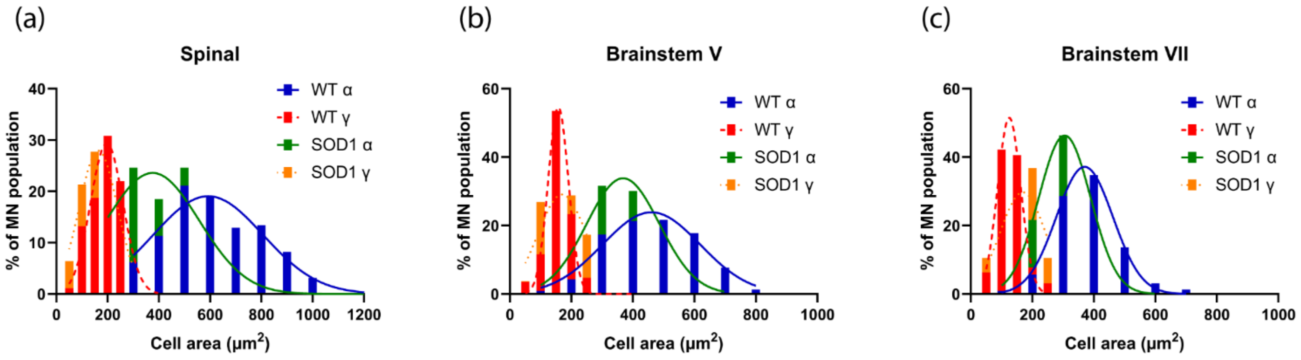


**Figure S4**. Analysis of motor neurons cell size distributions of α- and γ- subpopulations from WT and endstage SOD1$^{G93A}$ mice based on ChAT immunoreactivity in the (a) spinal cord, (b) brainstem cranial nerve V and (c) brainstem cranial nerve VII. Sizes are presented in frequency histograms using 50μm$^2$ bins for sizes under 300 μm$^2$ and 100 μm$^2$ bins for sizes greater than 300 μm$^2$. The data was fit to Gaussian curves for WT α-MNs (blue), WT γ-MNs (red), SOD1 α-MNs (green) and SOD1 γ-MNs (orange). The intersection of these normal distributions was used to determine the optimal size threshold separating α- and γ-MNs.

**Detailed Statistical Outcomes**

**Figure 1. T-test**

| Figure ID | Unpaired t-test | F-test of variance |
|---|---|---|
| 1d | 0.5096 | 0.7870 |
| 1e | 0.7955 | 0.9542 |
| 1i | 0.0112 | 0.3797 |
| 1j | 0.0105 | 0.1358 |

**Figure 1g. Two-way ANOVA**

| | | | | | |
|---|---|---|---|---|---|
| Two-way ANOVA | Ordinary | | | | |
| Alpha | 0.05 | | | | |
| | | | | | |
| Source of Variation | % of total variation | P value | P value summary | Significant? | |
| Interaction | 2.093 | 0.2369 | ns | No | |
| M1 region | 69.6 | <0.0001 | **** | Yes | |
| Genotype | 18.06 | 0.0056 | ** | Yes | |
| | | | | | |
| ANOVA table | SS | DF | MS | F (DFn, DFd) | P value |
| Interaction | 531640 | 1 | 531640 | F (1, 8) = 1.634 | P=0.2369 |
| M1 region | 17682636 | 1 | 17682636 | F (1, 8) = 54.36 | P<0.0001 |
| Genotype | 4589536 | 1 | 4589536 | F (1, 8) = 14.11 | P=0.0056 |
| Residual | 2602230 | 8 | 325279 | | |

| | | | | | |
|---|---|---|---|---|---|
| Compare each cell mean with the other cell mean in that row | | | | | |
| | | | | | |
| Number of families | 1 | | | | |
| Number of comparisons per famil | 2 | | | | |
| Alpha | 0.05 | | | | |
| | | | | | |
| Šídák's multiple comparisons test | Mean Diff. | 95.00% CI of diff. | Below threshold? | Summary | Adjusted F |
| | | | | | |
| WT - SOD1G93A | | | | | |
| II/III | 815.9 | -461.6 to 2093 | No | ns | 0.2218 |
| V | 1658 | 380.4 to 2935 | Yes | * | 0.0147 |
| | | | | | |
| II/III - V | | | | | |
| WT | 2007 | 729.4 to 3284 | Yes | ** | 0.0052 |
| SOD1G93A | 2849 | 1571 to 4126 | Yes | *** | 0.0006 |

**Figure 2. T-test**

| Figure ID | Unpaired t-test | F-test of variance |
|---|---|---|
| 2c | 0.9148 | 0.9524 |
| 2d | 0.0127 | 0.7907 |
| 2g | 0.9208 | 0.6848 |
| 2h | 0.0189 | 0.3273 |
| 2k | 0.1793 | 0.7221 |
| 2l | 0.3848 | 0.5452 |

**Figure 3. T-test**

| Figure ID | Unpaired t-test | F-test of variance |
|---|---|---|
| 3d | <0.0001 | 0.4568 |
| 3g | 0.0246 | 0.9737 |
| 3j | 0.0189 | 0.9600 |
| 3k | 0.0024 | 0.3365 |
| 3l | 0.7014 | 0.7453 |
| 3m | 0.0166 | 0.0701 |

**Figure 3c. Two-way ANOVA**

| | | | | | |
|---|---|---|---|---|---|
| Two-way ANOVA | Ordinary | | | | |
| Alpha | 0.05 | | | | |
| | | | | | |
| Source of Variation | % of total variation | P value | P value summary | Significant? | |
| Interaction | 8.1 | <0.0001 | **** | Yes | |
| MNsubtype | 72.52 | <0.0001 | **** | Yes | |
| Genotype | 14.33 | <0.0001 | **** | Yes | |
| | | | | | |
| ANOVA table | SS | DF | MS | F (DFn, DFd) | P value |
| Interaction | 685.3 | 1 | 685.3 | F (1, 20) = 32.11 | P<0.0001 |
| MNsubtype | 6136 | 1 | 6136 | F (1, 20) = 287.5 | P<0.0001 |
| Genotype | 1212 | 1 | 1212 | F (1, 20) = 56.81 | P<0.0001 |
| Residual | 426.8 | 20 | 21.34 | | |

| | | | | | |
|---|---|---|---|---|---|
| Compare each cell mean with the other cell mean in that row | | | | | |
| | | | | | |
| Number of families | 1 | | | | |
| Number of comparisons per family | 2 | | | | |
| Alpha | 0.05 | | | | |
| | | | | | |
| Šídák's multiple comparisons test | Mean Diff. | 95.00% CI of diff. | Below threshold? | Summary | Adjusted P [illegible] |
| | | | | | |
| WT - SOD1$^{G93A}$ | | | | | |
| α | 24.9 | 18.46 to 31.35 | Yes | **** | <0.0001 |
| γ | 3.528 | -2.919 to 9.974 | No | ns | 0.3614 |
| | | | | | |
| α - γ | | | | | |
| WT | 42.67 | 36.22 to 49.11 | Yes | **** | <0.0001 |
| SOD1$^{G93A}$ | 21.29 | 14.84 to 27.74 | Yes | **** | <0.0001 |

## Figure 3f. Two-way ANOVA

| | | | | | |
|---|---|---|---|---|---|
| Two-way ANOVA | Ordinary | | | | |
| Alpha | 0.05 | | | | |
| | | | | | |
| Source of Variation | % of total variation | P value | P value summary | Significant? | |
| Interaction | 2.128 | <0.0001 | **** | Yes | |
| MN subtype | 93.03 | <0.0001 | **** | Yes | |
| Genotype | 3.212 | <0.0001 | **** | Yes | |
| | | | | | |
| ANOVA table | SS | DF | MS | F (DFn, DFd) | P value |
| Interaction | 975.4 | 1 | 975.4 | F (1, 20) = 26.03 | P<0.0001 |
| MN subtype | 42645 | 1 | 42645 | F (1, 20) = 1138 | P<0.0001 |
| Genotype | 1473 | 1 | 1473 | F (1, 20) = 39.30 | P<0.0001 |
| Residual | 749.4 | 20 | 37.47 | | |

| | | | | | |
|---|---|---|---|---|---|
| Compare each cell mean with the other cell mean in that row | | | | | |
| | | | | | |
| Number of families | 1 | | | | |
| Number of comparisons per family | 2 | | | | |
| Alpha | 0.05 | | | | |
| | | | | | |
| Šídák's multiple comparisons test | Mean Diff. | 95.00% CI of diff. | Below threshold? | Summary | Adjusted P [illegible] |
| | | | | | |
| WT - SOD1$^{G93A}$ | | | | | |
| α | 28.42 | 19.87 to 36.96 | Yes | **** | <0.0001 |
| γ | 2.917 | -5.625 to 11.46 | No | ns | 0.6624 |
| | | | | | |
| α - γ | | | | | |
| WT | 97.06 | 88.51 to 105.6 | Yes | **** | <0.0001 |
| SOD1$^{G93A}$ | 71.56 | 63.01 to 80.10 | Yes | **** | <0.0001 |

## Figure 4. T-test

| Figure ID | Unpaired t-test | F-test of variance |
|---|---|---|
| 4c | 0.1009 | 0.3836 |
| 4d | 0.5192 | 0.3709 |
| 4e | 0.0192 | 0.1688 |
| 4g | 0.5312 | 0.1582 |
| 4i | 0.0019 | 0.9543 |
| 4l | 0.8440 | 0.8100 |
| 4m | 0.3037 | 0.7704 |

**Figure 5. T-test**

| Figure ID | Unpaired t-test | F-test of variance |
|---|---|---|
| 5c | <0.0001 | 0.3728 |
| 5g | 0.0281 | 0.2913 |

**Figure 5f. Two-way ANOVA**

| | | | | | |
|---|---|---|---|---|---|
| Two-way ANOVA | Ordinary | | | | |
| Alpha | 0.05 | | | | |
| | | | | | |
| Source of Variation | % of total variation | P value | P value summary | Significant? | |
| Interaction | 20.75 | <0.0001 | **** | Yes | |
| MN subtype | 54.01 | <0.0001 | **** | Yes | |
| Genotype | 25.26 | <0.0001 | **** | Yes | |
| | | | | | |
| ANOVA table | SS (Type III) | DF | MS | F (DFn, DFd) | P value |
| Interaction | 365.7 | 1 | 365.7 | F (1, 18) = 68.00 | P<0.0001 |
| MN subtype | 952 | 1 | 952 | F (1, 18) = 177.0 | P<0.0001 |
| Genotype | 445.3 | 1 | 445.3 | F (1, 18) = 82.80 | P<0.0001 |
| Residual | 96.8 | 18 | 5.378 | | |

| | | | | | |
|---|---|---|---|---|---|
| Compare each cell mean with the other cell mean in that row | | | | | |
| | | | | | |
| Number of families | 1 | | | | |
| Number of comparisons per family | 2 | | | | |
| Alpha | 0.05 | | | | |
| | | | | | |
| Šídák's multiple comparisons test | Predicted (LS) | 95.00% CI of diff. | Below threshold? | Summary | Adjusted P [illegible] |
| | | | | | |
| WT - $SOD1^{G93A}$ | | | | | |
| α | 17.22 | 13.80 to 20.65 | Yes | **** | <0.0001 |
| γ | 0.8473 | -2.577 to 4.272 | No | ns | 0.8009 |
| | | | | | |
| α - γ | | | | | |
| WT | 21.4 | 17.82 to 24.98 | Yes | **** | <0.0001 |
| $SOD1^{G93A}$ | 5.023 | 1.757 to 8.288 | Yes | ** | 0.0029 |

**Figure 6. T-test**

| Figure ID | Unpaired t-test | F-test of variance |
|---|---|---|
| 6c | 0.1808 | 0.0548 |
| 6g | 0.0404 | 0.0612 |
| 6m | 0.1989 | 0.1148 |
| 6n | 0.0005 | 0.1923 |
| 6o | 0.0709 | 0.2470 |
| 6p | 0.0023 | 0.6787 |

**Figure 6f. Two-way ANOVA**

| | | | | | |
|---|---|---|---|---|---|
| Two-way ANOVA | Ordinary | | | | |
| Alpha | 0.05 | | | | |
| | | | | | |
| Source of Variation | % of total variation | P value | P value summary | Significant? | |
| Interaction | 34.4 | <0.0001 | **** | Yes | |
| MNsubtype | 25.44 | <0.0001 | **** | Yes | |
| Genotype | 29.59 | <0.0001 | **** | Yes | |
| | | | | | |
| ANOVA table | SS (Type III) | DF | MS | F (DFn, DFd) | P value |
| Interaction | 245.4 | 1 | 245.4 | F (1, 14) = 151.8 | P<0.0001 |
| MNsubtype | 181.5 | 1 | 181.5 | F (1, 14) = 112.3 | P<0.0001 |
| Genotype | 211.1 | 1 | 211.1 | F (1, 14) = 130.6 | P<0.0001 |
| Residual | 22.64 | 14 | 1.617 | | |

| | | | | | |
|---|---|---|---|---|---|
| Compare each cell mean with the other cell mean in that row | | | | | |
| | | | | | |
| Number of families | 1 | | | | |
| Number of comparisons per family | 2 | | | | |
| Alpha | 0.05 | | | | |
| | | | | | |
| Šídák's multiple comparisons test | Predicted (LS) | 95.00% CI of diff. | Below threshold? | Summary | Adjusted P |
| | | | | | |
| WT - SOD1$^{G93A}$ | | | | | |
| α | 14.32 | 12.19 to 16.46 | Yes | **** | <0.0001 |
| γ | -0.5385 | -2.673 to 1.597 | No | ns | 0.7866 |
| | | | | | |
| α - γ | | | | | |
| WT | 13.82 | 11.81 to 15.83 | Yes | **** | <0.0001 |
| SOD1$^{G93A}$ | -1.041 | -3.291 to 1.210 | No | ns | 0.4619 |

**Figure 7. T-test**

| Figure ID | Unpaired t-test | F-test of variance |
|---|---|---|
| 7e | 0.6794 | 0.9430 |

**Figure 7b. Two-way ANOVA**

| | | | | | |
|---|---|---|---|---|---|
| Two-way ANOVA | Ordinary | | | | |
| Alpha | 0.05 | | | | |
| | | | | | |
| Source of Variation | % of total variation | P value | P value summary | Significant? | |
| Interaction | 18.33 | 0.0001 | *** | Yes | |
| Time | 43.43 | <0.0001 | **** | Yes | |
| Genotype | 20.64 | <0.0001 | **** | Yes | |
| | | | | | |
| ANOVA table | SS (Type III) | DF | MS | F (DFn, DFd) | P value |
| Interaction | 37.78 | 1 | 37.78 | F (1, 18) = 24.08 | P=0.0001 |
| Time | 89.48 | 1 | 89.48 | F (1, 18) = 57.04 | P<0.0001 |
| Genotype | 42.53 | 1 | 42.53 | F (1, 18) = 27.11 | P<0.0001 |
| Residual | 28.24 | 18 | 1.569 | | |

| | | | | | |
|---|---|---|---|---|---|
| Compare each cell mean with the other cell mean in that row | | | | | |
| | | | | | |
| Number of families | 1 | | | | |
| Number of comparisons per family | 2 | | | | |
| Alpha | 0.05 | | | | |
| | | | | | |
| Šídák's multiple comparisons test | Predicted (LS) | 95.00% CI of diff. | Below threshold? | Summary | Adjusted P [illegible] |
| | | | | | |
| WT - SOD1 | | | | | |
| P60 | 0.1607 | -1.771 to 2.093 | No | ns | 0.9749 |
| P120 | 5.424 | 3.661 to 7.188 | Yes | **** | <0.0001 |
| | | | | | |
| P60 - P120 | | | | | |
| WT | 1.419 | -0.4312 to 3.268 | No | ns | 0.1495 |
| SOD1 | 6.682 | 4.832 to 8.532 | Yes | **** | <0.0001 |

## Figure 7c. Two-way ANOVA

| | | | | | |
|---|---|---|---|---|---|
| Two-way ANOVA | Ordinary | | | | |
| Alpha | 0.05 | | | | |
| | | | | | |
| Source of Variation | % of total variation | P value | P value summary | Significant? | |
| Interaction | 0.0358 | 0.891 | ns | No | |
| Time | 5.538 | 0.1011 | ns | No | |
| Genotype | 60.81 | <0.0001 | **** | Yes | |
| | | | | | |
| ANOVA table | SS (Type III) | DF | MS | F (DFn, DFd) | P value |
| Interaction | 0.004105 | 1 | 0.004105 | F (1, 18) = 0.0193 | P=0.8910 |
| Time | 0.635 | 1 | 0.635 | F (1, 18) = 2.987 | P=0.1011 |
| Genotype | 6.973 | 1 | 6.973 | F (1, 18) = 32.80 | P<0.0001 |
| Residual | 3.827 | 18 | 0.2126 | | |

| | | | | | |
|---|---|---|---|---|---|
| Compare each cell mean with the other cell mean in that row | | | | | |
| | | | | | |
| Number of families | 1 | | | | |
| Number of comparisons per family | 2 | | | | |
| Alpha | 0.05 | | | | |
| | | | | | |
| Šídák's multiple comparisons test | Predicted (LS) | 95.00% CI of diff. | Below threshold? | Summary | Adjusted P |
| | | | | | |
| WT - SOD1G93A | | | | | |
| P60 | 1.158 | 0.4469 to 1.869 | Yes | ** | 0.0018 |
| P120 | 1.103 | 0.4540 to 1.752 | Yes | ** | 0.0012 |

## Figure 7d. Two-way ANOVA

| | | | | | |
|---|---|---|---|---|---|
| Two-way ANOVA | Ordinary | | | | |
| Alpha | 0.05 | | | | |
| | | | | | |
| Source of Variation | % of total variation | P value | P value summary | Significant? | |
| Interaction | 0.04264 | 0.889 | ns | No | |
| Time | 12.8 | 0.0246 | * | Yes | |
| Genotype | 48.71 | 0.0001 | *** | Yes | |
| | | | | | |
| ANOVA table | SS (Type III) | DF | MS | F (DFn, DFd) | P value |
| Interaction | 2.241 | 1 | 2.241 | F (1, 18) = 0.0200[illegible] | P=0.8890 |
| Time | 673 | 1 | 673 | F (1, 18) = 6.016 | P=0.0246 |
| Genotype | 2560 | 1 | 2560 | F (1, 18) = 22.89 | P=0.0001 |
| Residual | 2014 | 18 | 111.9 | | |

| | | | | | |
|---|---|---|---|---|---|
| Compare each cell mean with the other cell mean in that row | | | | | |
| | | | | | |
| Number of families | 1 | | | | |
| Number of comparisons per family | 2 | | | | |
| Alpha | 0.05 | | | | |
| | | | | | |
| Šídák's multiple comparisons test | Predicted (LS) | 95.00% CI of diff. | Below threshold? | Summary | Adjusted P [illegible] |
| | | | | | |
| WT - SOD1 | | | | | |
| P60 | 22.31 | 5.993 to 38.62 | Yes | ** | 0.0074 |
| P120 | 21.02 | 6.133 to 35.92 | Yes | ** | 0.0058 |
| | | | | | |
| P60 - P120 | | | | | |
| WT | 11.75 | -3.871 to 27.37 | No | ns | 0.1594 |
| SOD1 | 10.47 | -5.153 to 26.09 | No | ns | 0.2248 |
